# Autophagic degradation of aquaporin-2 is an early event in hypokalemia-induced nephrogenic diabetes insipidus

**DOI:** 10.1038/srep18311

**Published:** 2015-12-17

**Authors:** Sookkasem Khositseth, Panapat Uawithya, Poorichaya Somparn, Komgrid Charngkaew, Nattakan Thippamom, Jason D. Hoffert, Fahad Saeed, D. Michael Payne, Shu-Hui Chen, Robert A. Fenton, Trairak Pisitkun

**Affiliations:** 1 Department of Pediatrics, Faculty of Medicine, Thammasat University Klong Luang, Pathumthani, 12120, Thailand; 2 Department of Physiology, Faculty of Medicine Siriraj Hospital, Mahidol University Bangkok, 10700, Thailand; 3 Systems Biology Center, Research Affairs, Faculty of Medicine, Chulalongkorn University 1873 Rama 4 Road, Pathumwan, Bangkok, 10330, Thailand; 4 Department of Pathology, Faculty of Medicine Siriraj Hospital, Mahidol University Bangkok, 10700, Thailand; 5 National Institute of Diabetes and Digestive and Kidney, Bethesda MD 20892, United States; 6 Department of Electrical & Computer Engineering and Department of Computer Science, Western Michigan University Kalamazoo, 49008, United States; 7 Department of Chemistry, National Cheng Kung University, Tainan City, 701, Taiwan; 8 Department of Biomedicine and Center for Interactions of Proteins in Epithelial Transport, Aarhus University, Aarhus, 8000, Denmark; 9 Epithelial Systems Biology Laboratory, National Heart, Lung, and Blood Institute, Bethesda MD 20892, United States

## Abstract

Hypokalemia (low serum potassium level) is a common electrolyte imbalance that can cause a defect in urinary concentrating ability, i.e., nephrogenic diabetes insipidus (NDI), but the molecular mechanism is unknown. We employed proteomic analysis of inner medullary collecting ducts (IMCD) from rats fed with a potassium-free diet for 1 day. IMCD protein quantification was performed by mass spectrometry using a label-free methodology. A total of 131 proteins, including the water channel AQP2, exhibited significant changes in abundance, most of which were decreased. Bioinformatic analysis revealed that many of the down-regulated proteins were associated with the biological processes of generation of precursor metabolites and energy, actin cytoskeleton organization, and cell-cell adhesion. Targeted LC-MS/MS and immunoblotting studies further confirmed the down regulation of 18 selected proteins. Electron microscopy showed autophagosomes/autophagolysosomes in the IMCD cells of rats deprived of potassium for only 1 day. An increased number of autophagosomes was also confirmed by immunofluorescence, demonstrating co-localization of LC3 and Lamp1 with AQP2 and several other down-regulated proteins in IMCD cells. AQP2 was also detected in autophagosomes in IMCD cells of potassium-deprived rats by immunogold electron microscopy. Thus, enhanced autophagic degradation of proteins, most notably including AQP2, is an early event in hypokalemia-induced NDI.

Hypokalemia (low serum potassium level) is a very common electrolyte imbalance encountered in clinical medicine, occurring as a result of poor nutritional status, gastrointestinal diseases, and side effects from numerous medications. This potentially life-threatening condition adversely affects multiple organ systems, producing cardiac arrhythmias, muscle weakness and various abnormalities in renal function. One of the renal impairments caused by hypokalemia is a reduction in urinary concentrating ability and a lack of response to the antidiuretic hormone arginine vasopressin (AVP), resulting in nephrogenic diabetes insipidus (NDI; characterized by excessive thirst and excretion of large amounts of very dilute urine).

The ability to maintain water balance in humans is based on the function of kidneys to concentrate urine, a process which is regulated through the vasopressin/V2-receptor/aquaporin-2 axis. Approximately 180 L/day of blood entering the kidneys is filtered through glomeruli, however, only less than 1% of the filtered water is finally excreted as urine. In the kidneys, 90% of the filtered fluid is reabsorbed in the proximal tubules and thin ascending limb of Henle’s loop. The remaining 10% of the filtered fluid is reabsorbed in the connecting tubules and collecting ducts under the control of vasopressin, the water channel protein aquaporin-2 (AQP2), and other signal molecules^1^,^2^. The collecting duct system plays a critical role in fluid homeostasis since it is the site for the final stage of the urine concentrating process.

The ability of the collecting duct to concentrate urine depends predominantly on the functions of key proteins involved in two interdependent processes. First, the NKCC2 protein (bumetanide-sensitive Na^+^-K^+^-2Cl^−^ cotransporter) functions to generate medullary interstitial hyperosmolality. Second, AQP2 and urea transporter proteins (UT-A1, UT-A3) regulate osmotic water and urea permeability of the renal collecting duct epithelium. AQP2 is expressed in principal cells of the cortical, outer, and inner medullary collecting ducts and is abundant in both the apical plasma membrane and subapical vesicles^3^. AQP2 is the primary target for vasopressin regulation of collecting duct water permeability^4^. The short-term regulation of AQP2 by vasopressin occurs in a period of minutes, accompanied by phosphorylation at serine 256 of AQP2, resulting in trafficking of AQP2-containing vesicles to the apical membrane and increasing water permeability of the principal cell^5^,^6^,^7^. The long-term vasopressin regulation, occurring over a period of hours to days, increases whole-cell abundance of AQP2^8^,^9^,^10^.

Potassium deprivation has been reported to induce urinary concentrating defects through alterations in abundances of various proteins, including NKCC2 in thick ascending limb cells as well as AQP2 and/or urea transporter proteins in inner medullary collecting duct cells^11^,^12^,^13^,^14^. However, even though the induction of NDI by hypokalemia has been known for decades^15^, the mechanism remains a long-standing mystery.

While most studies of hypokalemia-induced NDI have focused on long-term effects (days to weeks) in the collecting duct, the onset of a urinary concentrating defect in potassium-deprived rats has been reported to be as early as 12–24 hours^13^. In order to capture the initial alterations that might lead to the pathogenesis of hypokalemia-induced NDI, we chose to investigate the proteome of the collecting duct from potassium-deprived rats at a time before a significant urinary concentrating defect and the consequent confounding alterations have already occurred. Since the inner medullary collecting duct (IMCD) is an important part of the collecting duct system and can be easily isolated in large amounts with high purity, it is more suitable for proteomic experiments than other parts of the collecting duct. Thus, mass spectrometry-based proteomics was employed to identify and quantify changes in abundance of IMCD proteins in this study. The proteomic findings revealed a reduction in abundance of AQP2, as well as proteins involved in mitochondrial energy metabolism, actin cytoskeleton organization, and cell-cell adhesion. The decreased abundance of these proteins was further confirmed by targeted liquid chromatography-tandem mass spectrometry (LC-MS/MS) and immunoblotting.

Next, in an extension of our proteomic analysis, we employed both electron microscopy (EM) and immunofluorescence labeling of IMCD cells to identify the possible mechanism for the observed protein down-regulation. Both of these analyses revealed that the decreased abundance of identified proteins was related to autophagic protein degradation. Numerous studies have demonstrated induction of autophagy in kidney diseases^16^ and kidney tubular injury resulting from various conditions, e.g., nephrotoxins^17^ and ischemic injury^18^. This study is the first to report that autophagy is an early event involved in a common electrolyte imbalance-induced NDI.

## Results

### Animal model of hypokalemia-induced NDI

**Figure 1:**
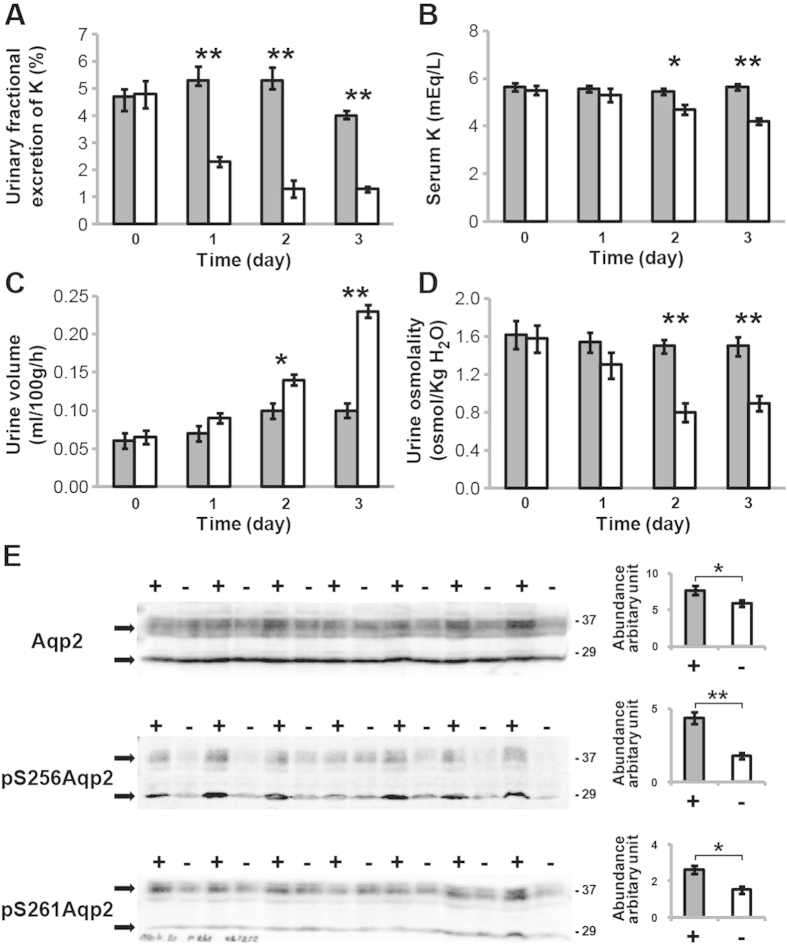
Physiological parameters of a rat model of early hypokalemia-induced NDI. Urinary fractional excretion of potassium (**A**), serum potassium (**B**), urine volume (**C**), and urine osmolality (**D**) were measured in rats receiving either a normal chow diet (■, n = 7) or a potassium-free diet (NK; □, n = 7) for 1, 2, and 3 days. The units for urinary fractional excretion of potassium (shown as %) are a ratio of urinary:plasma potassium concentration normalized to that of urinary:plasma creatinine concentration. (**E**) Immunoblotting of inner medullary proteins from control rats (+, ■, n = 7) and potassium-deprived rats (–, □, n = 7) after 1 day of potassium deprivation. The two bands of AQP2 (29 kDa non-glycosylated and 37 kDa glycosylated isoforms) are indicated with arrows.

Rats were fed a potassium-free diet (NK) or normal chow diet for 1–3 days with monitoring of key physiological parameters. The earliest detectable change was in the urinary fractional excretion of potassium, which decreased significantly (59%) (*P* < 0.01) within 24 hours of initiating the potassium-free diet (Fig. 1A). For the other monitored parameters, significant changes of serum potassium (*P* < 0.05), urine volume (*P* < 0.05), and urine osmolality (*P* < 0.01) were detected by day 2, representing the earliest appearance of hypokalemia and a urinary concentrating defect (Fig. 1B–D). Decreased urinary excretion of potassium preceding the onset of hypokalemia is explained by the feedforward homeostatic control mechanism involving the gut dietary potassium sensor as described previously^19^,^20^,^21^ (see Discussion for further details).

Immunoblotting of proteins from the entire inner medulla demonstrated a 23% decrease in total AQP2 protein (*P* < 0.05) after 1 day of potassium deprivation (Fig. 1E), in agreement with previous studies^12^,^13^. As an additional novel finding in the present study, even larger decreases were detected for phosphorylation of AQP2 at both vasopressin-sensitive sites^22^, pS256 (59.6%) and pS261 (43.4%). The decreases in AQP2 protein abundance and phosphorylation at day 1 should have been accompanied by an early urinary concentration defect (i.e., reflected by a decrease in urine osmolality), which was not actually detected until day 2 (Fig. 1D). However, since the urine osmolality was an average value from the 24-h collection, it did not represent the actual urine osmolality at the final hours of each collecting period. When urine osmolality was measured in urine recovered from bladders of some of the potassium-deprived rats at 24 hours (i.e., from those animals that had full bladders during kidney tissue harvesting), lower urine osmolality was detected compared to the controls (Supplementary Fig. 1), suggesting that a urinary concentrating defect was present near the end of day 1 in those animals. Cyclic adenosine monophosphate (cAMP) levels in IMCD cells were not significantly different between the potassium-deprived and control rats after 1 day (NK: 8.6 ± 1.0 *vs.* Control: 10.7 ± 0.52 fmol/μg, *P* > 0.05). These results confirmed that, even before the appearance of frank hypokalemia, early molecular changes occurred in IMCD cells after only 1 day of potassium deprivation.

### Identification of global molecular changes by proteomics and bioinformatics

**Figure 2:**
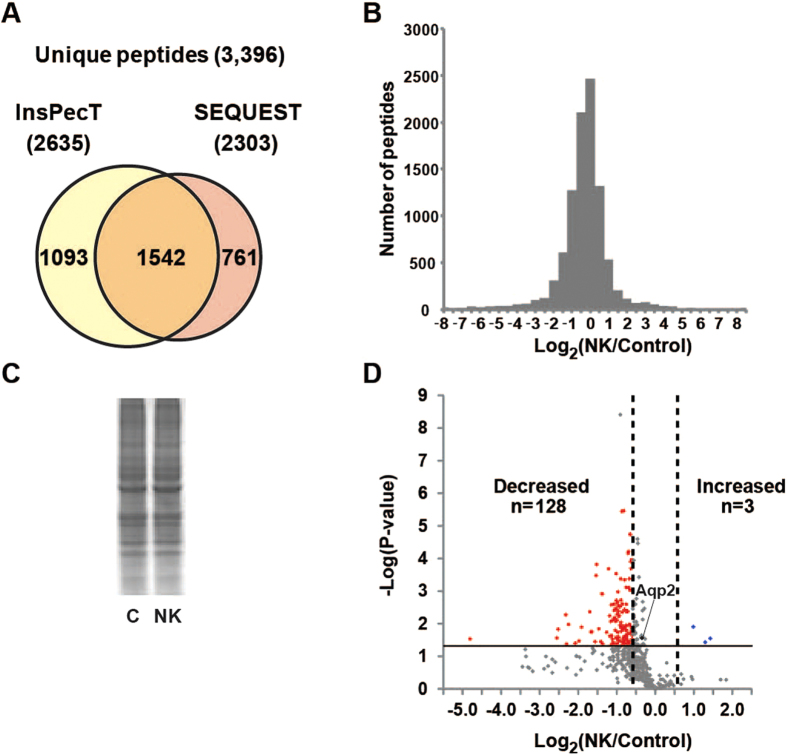
Proteomic analysis of IMCD proteins from the potassium-deprived and control rats. (**A**) A total of 3,396 unique peptides were identified. (**B**) Distribution of log_2_peptide abundance ratios between two groups for all quantified peptides. (**C**) A Coomassie-stained gel demonstrates an equal loading amount of total IMCD proteins from the two groups for the proteomic analysis. (**D**) A volcano plot of 475 proteins observed in all three biological replicates. A horizontal solid line indicates *P*-value of 0.05 [−log(*P*-value) of 1.3]. Vertical dashed lines indicate 95% CI of protein abundance ratios [|log_2_(NK/Control)| ≥ 0.58]^25^).

For proteomic analysis, we selected a time point of 1 day after initiation of potassium deprivation, the time at which the initial molecular changes had started to occur without any change in serum potassium level. A total of 3,396 unique peptides, 2,303 from SEQUEST and 2,635 from InsPecT (Fig. 2A), were identified in IMCD cells with a false discovery rate (FDR) of <1% using a target-decoy analysis (Supplementary data 1). A total of 2,368 unambiguous peptides (i.e., those matching a single protein) were assigned to 802 proteins by the ProMatch program^23^. The quantification of unambiguous peptides was determined from the extracted-ion chromatograms using QUOIL software^24^. A histogram of the base-2 logarithm of abundance ratios for all quantified peptides between the potassium-deprived and control groups (Fig. 2B) reveals that the abundances of the majority of peptides were not changed substantially, as confirmed by the total protein intensities in a representative coomassie-stained gel (Fig. 2C). However, a slightly left-skewed distribution (skewness = −0.12, *P* < 0.05) of the histogram is also observed, reflecting a slight tendency toward decreased abundance among the total group of quantified peptides. Relative abundance at the protein level was then calculated for each protein based on the median abundance ratio of the corresponding peptides in control and potassium-depleted states (Supplementary data 2). A volcano plot in Fig. 2D illustrates the average log_2_ protein abundance ratios (of those proteins observed in all three biological replicates, total of 475 proteins) versus significance, expressed in –log_10_ (*P*-value). Proteins exhibiting significant changes in abundance were identified using a dual statistical criterion: (i) *P* < 0.05 [−log_10_ (*P*-value) > 1.3] by two-tailed t-test and (ii) average |log_2_ (NK/Control)| ≥ 0.58 (based on a reported 95% confidence interval [95% CI] of the label-free LC-MS/MS technique^25^). Based on this stringent analysis, we identified 128 proteins that significantly decreased in abundance, while only 3 proteins were significantly increased in IMCD cells of potassium-deprived rats (Supplementary tables 1 and 2). Note that AQP2 was significantly decreased by 28% in this proteomic experiment, in agreement with the previous immunoblotting result shown in Fig. 1E, and confirming the early response to potassium deprivation. A complete list of protein identification, protein quantification, and associated Gene Ontology (GO) terms from this study is provided in a publicly accessible online database at https://hpc.nih.gov/ESBL/Database/HypoK/.

**Figure 3:**
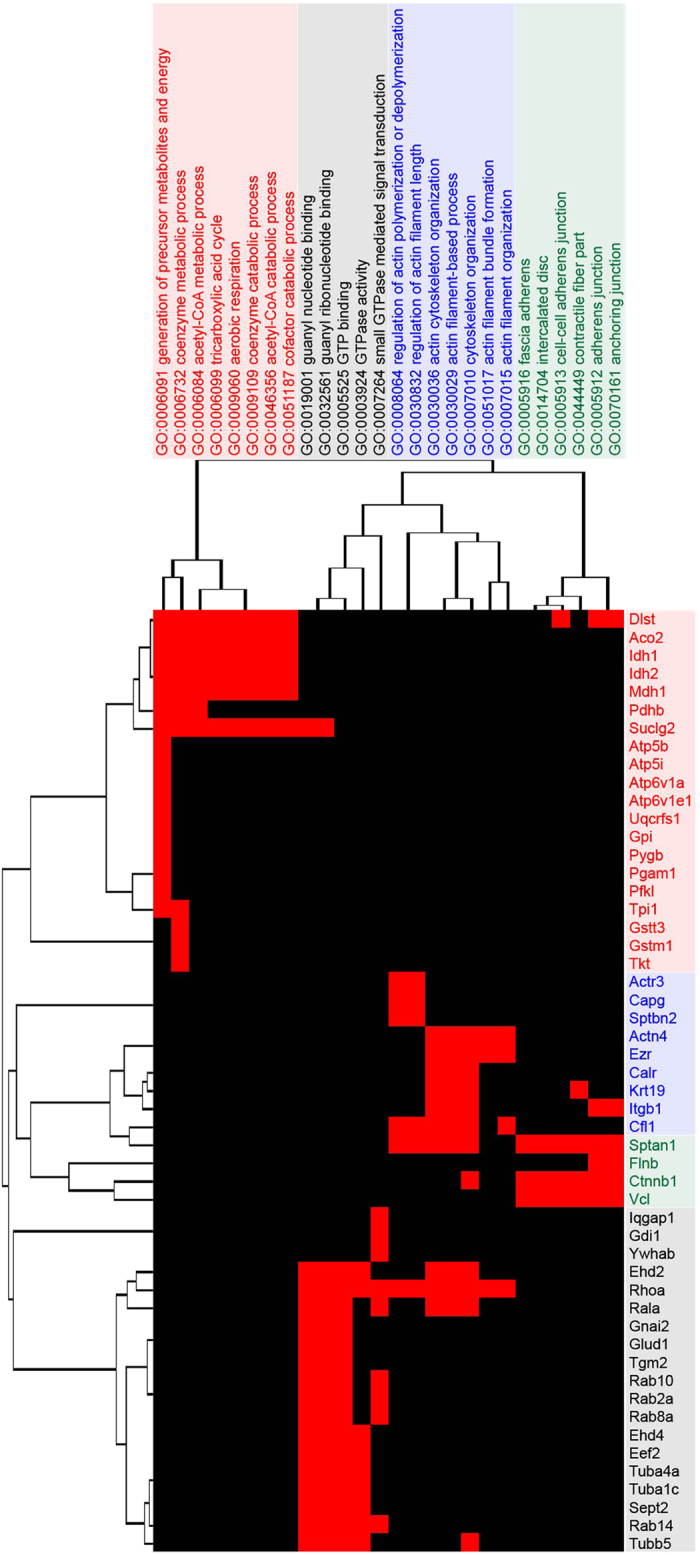
Highly-represented functional annotation clusters of the significantly changed proteins. Vertical dendrogram demonstrates four over-represented clusters (*P* < 0.05) of GO terms. Horizontal dendrogram shows the list of proteins for each cluster.

The proteins exhibiting significant changes in abundance before the onset of hypokalemia-induced NDI were further investigated using the DAVID bioinformatics resource^26^. The highly-represented annotations were grouped into clusters (using a list of all IMCD-expressed transcripts as a background). The most significantly enriched clusters included proteins with the functional GO terms “generation of precursor metabolites and energy”, “guanyl nucleotide binding”, “actin cytoskeleton organization”, and “fascia adherens” (Fig. 3 and Supplementary table 3).

### Data verification by targeted LC-MS/MS and immunoblotting

In order to confirm the observed findings from the proteomic and bioinformatic analyses, a targeted LC-MS/MS approach was utilized as an independent measurement. A new animal experiment was performed for this study using the same upstream experimental setup as for the non-targeted LC-MS/MS experiments. A list of protein names, peptide sequences, precursor m/z ratios, retention times, and integrated peak areas of the 16 proteins selected for analysis by targeted LC-MS/MS is provided in Supplementary data 3.

Consistent with the non-targeted LC-MS/MS technique, the targeted analysis confirmed a reduction in the abundances of all the selected proteins from the discovery proteomic studies (Supplementary Fig. 2A). For instance, we validated down-regulation of 9 proteins in the “generation of precursor metabolites and energy” cluster (Atp6v1e1, Atp5b, Atp6v1a, Atp5i, Ndufs3, Aco2, Idh1, Idh2, and Mdh1), all of which are known mitochondrial proteins. (Beyond the mentioned cluster, there were 22 more mitochondrial proteins that were identified as significantly decreased by the non-targeted LC-MS/MS studies.) Down-regulation was also confirmed for the major proteins in the “actin cytoskeleton organization” and “fascia adherens” clusters (Itgb1, Actn4, Capg, Ctnnb1, Ezr, Sptan1, and Vcl). In addition to the targeted LC-MS/MS results, immunoblotting confirmed the significant down-regulation of three proteins in the “fascia adherens” and “guanyl nucleotide binding” clusters [β-catenin (Ctnnb1) by 30.3%, cortactin (Cttn) by 88.7%, and RhoA by 25%, respectively) in IMCD cells of potassium-deprived rats (Supplementary Fig. 2B).

**Figure 4:**
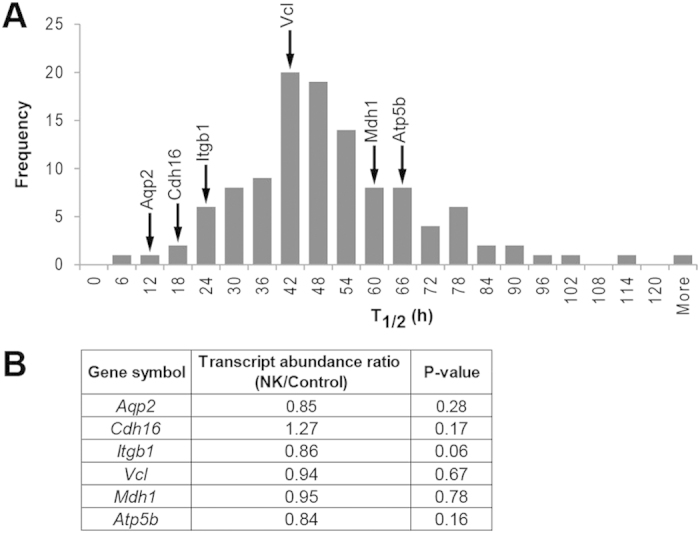
Estimated half-lives and transcript quantification of down-regulated proteins. (**A**) A distribution of the estimated half-lives for the down-regulated proteins identified in this proteomic study (based on mouse collecting duct proteins^27^). The estimated half-lives of AQP2 and five representative proteins are indicated by arrows (**A**) and their transcript abundance ratios (NK/Control) as determined by qRT-PCR are shown (**B**).

The early decline (after only 1 day of potassium deprivation) in the abundance of the proteins identified by proteomic analysis could be explained by either an increase in protein degradation or a reduction in protein production of short half-life proteins (i.e., t_1/2_ shorter than 24 hours). Based on cross-referencing with the t_1/2_ values at basal conditions for the mouse collecting duct proteins as reported by Sandoval *et al.*^27^, the majority of the down-regulated proteins was estimated to possess t_1/2_ longer than 24 hours (Fig. 4A and Supplementary table 4), thereby eliminating a decrease in protein production as the likely mechanism for the down-regulation of the majority of identified proteins. However, AQP2 and some of the other down-regulated proteins (e.g., Cdh16 and Itgb1) have reported half-lives shorter than 24 hours. Therefore, we quantified changes in mRNA levels of *Aqp2* and five representative transcripts from the highly-represented GO clusters (*Cdh16*, *Itgb1*, *Vcl, Mdh1*, and *Atp5b*). Figure 4B demonstrates no significant differences between the potassium-deprived and control groups for these six transcripts, indicating that the observed decreases in abundance of these proteins are not regulated at the transcriptional level. Thus, other post-transcriptional processes, such as decreased rate of translation and/or increased protein degradation, are responsible for the observed reductions in protein abundance.

### Structural abnormalities of IMCD in early hypokalemia-induced NDI

**Figure 5:**
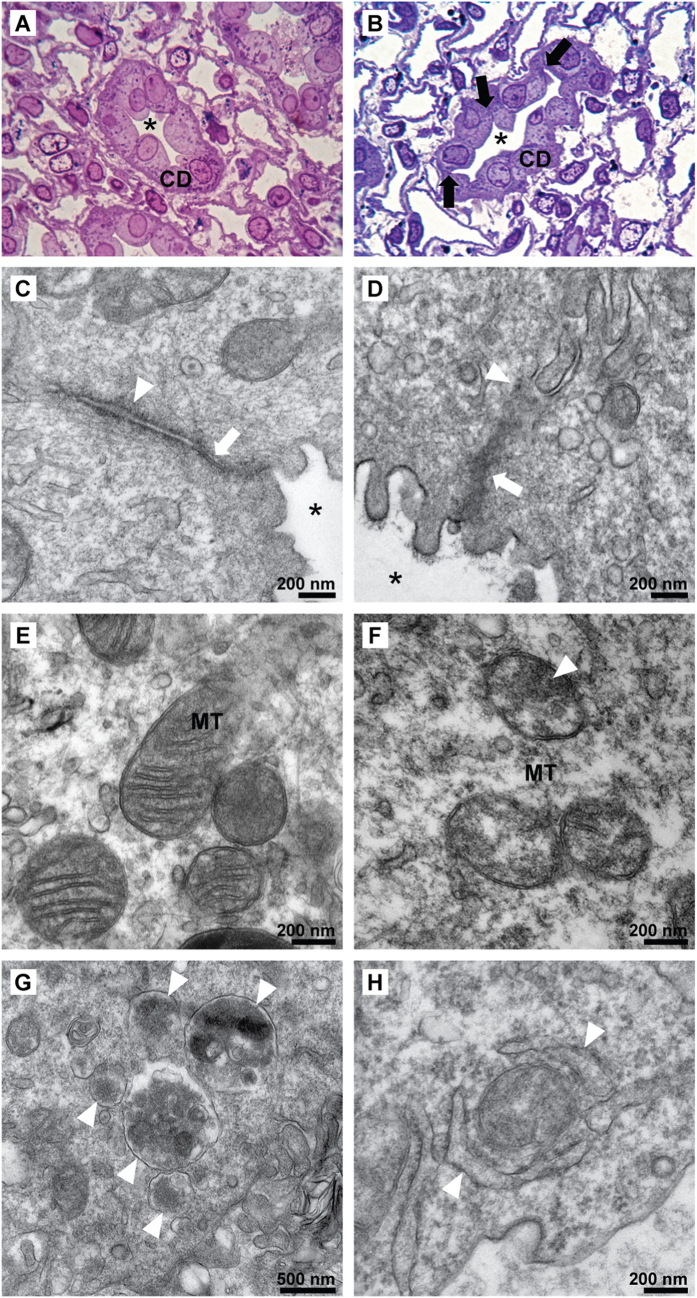
Structural abnormalities of IMCD in early potassium-deprived rats. Compared to control IMCD (***A***), histopathology in potassium-deprived IMCD (**B**) shows disruption between IMCD cells (arrows) and a collapsed tubular lumen (*), Toluidine Blue, original magnification 400x. (**C**) An electron micrograph (EM) of the intercellular junction of control IMCD cells demonstrates a thin tight junction (arrow) and thin delicate adherens junction (arrow head). (**D**) A tight junction with disorganized filaments as well as thick electron dense deposition (arrow) and ill-defined adherens junction with thick electron dense deposition (arrow head) were observed in potassium-deprived IMCD cells. (***E***) Normal mitochondria in control IMCD cells. (**F**) Abnormal mitochondria in potassium-deprived IMCD show swollen cristae with the focal electron dense deposition (arrow head). Autophagolysosomes (**G**, arrow heads) and mitophagy (**H**, arrow heads) in IMCD cells of the potassium-deprived rats.

To elucidate the possible mechanism for the protein down-regulation identified by proteomic analysis, we first performed structural and ultrastructural analyses of the IMCD after 1 day of potassium deprivation. Histopathology of the IMCD showed disruption between tubular cells, and occasional collapsed tubules in the potassium-deprived conditions (Fig. 5B *vs*. the control in 5A). The observed changes were widespread, occurring in all of the examined fields. Electron micrographs revealed distinct ultrastructural changes in the intercellular junctions of the IMCD from potassium-deprived rats (Fig. 5D *vs.* the control in 5C), including a thick electron dense deposition and disorganized filaments at tight junctions. These observed ultrastructural changes were also widespread, occurring in all cells examined. Moreover, less distinct zonula adherens with thick electron dense deposition was observed in some cells. Electron micrographs of the IMCD from potassium-deprived rats also revealed electron dense depositions within the mitochondrial matrix and swelling of cristae in the majority of mitochondria in all cells (Fig. 5F), as compared to well-defined, sharp mitochondrial cristae in control IMCD (Fig. 5E). Strikingly, single-membrane autophagolysosomes containing electron dense membrane structures (Fig. 5G) and double-membrane autophagosome vesicles or phagophores (early stage of autophagosome formation) were identified by electron microscopy in every observed IMCD cell of the potassium-deprived rats; there were 2–3 autophagosomes/autophagolysosomes per cell, compared to 1 of these structures per 10 cells in control rats (*P* < 0.05). Damaged mitochondria engulfed by autophagosomes (mitophagy) (Fig. 5H) were found occasionally (but was not quantified) in the potassium-deprived group, however mitophagy was not seen in the control group. The structures of the endoplasmic reticulum, Golgi apparatus, and other organelles were not detectably affected. All findings were based on the observation of at least 10 random cells (electron microscopy) or microscopic fields (light microscopy) from two potassium-deprived rats and two control rats. The swelling and other structural changes to mitochondria we observed could be associated with an activation of apoptosis, which could also be responsible for the down-regulation of proteins identified in this study. To investigate a potential activation of apoptosis in response to potassium deprivation, we performed immunoblotting of both intact and cleaved forms of caspase 3 (Supplementary Fig. 3). The result demonstrated no statistically significant increase in the cleaved form of caspase 3, indicating that apoptosis does not seem to play a major role in the protein degradation process found in this study.

### Co-localization of the down-regulated proteins with autophagosomes/autophagolysosomes

**Figure 6:**
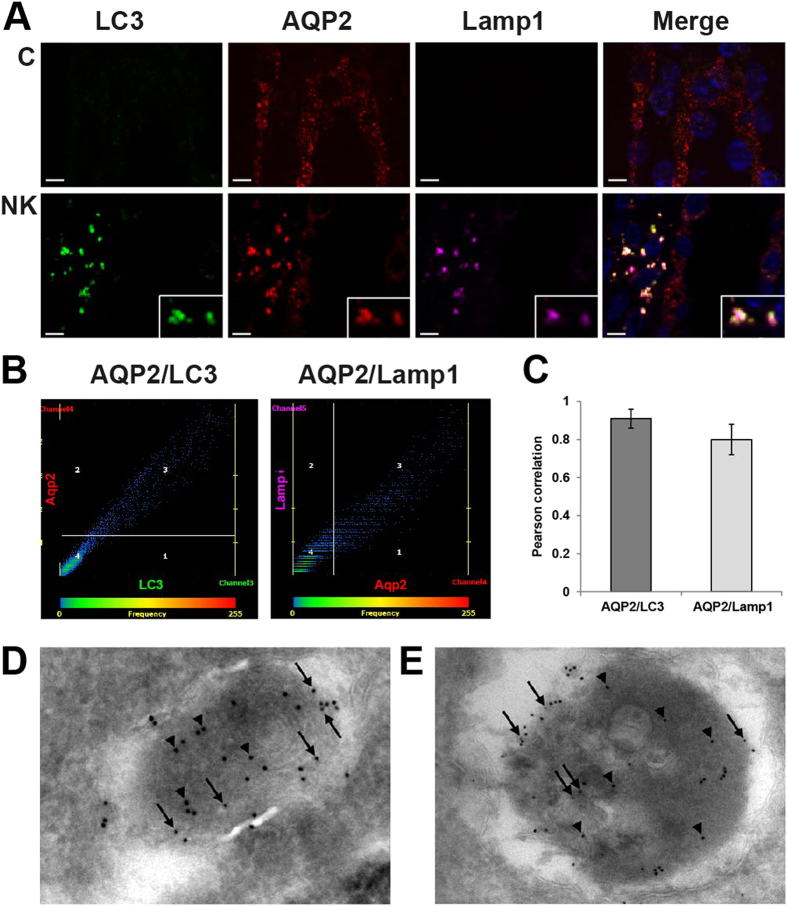
Co-localization of AQP2 with autophagy markers. (**A**) The inner medulla sections of control rats (C) and rats fed with a potassium-free diet for 1 day (NK) were triple-labeled against AQP2 (red), Lamp1 (pink), and LC3 (green). Insets demonstrate significant co-localization as a group of puncta, which were not observed in control sections. Scale bar = 4 μm. (**B**) Representative scatter plots demonstrate the high degree of co-localization between AQP2 and LC3 (r^2^ = 0.83, *P* < 0.05) or Lamp1 (r^2^ = 0.73, *P* < 0.05) after potassium deprivation. (**C**) A bar graph summarizes Pearson correlation coefficients of AQP2 and LC3 or Lamp1. (**D**) By immunogold EM, AQP2 (small gold particles, arrows) and LC3β (large gold particles, arrow heads) localize in autophagosomes in IMCD cells of potassium-deprived rats. (**E**) AQP2 (small gold particles, arrows) and cathepsin-D (large gold particles, arrow heads) also localize in similar membrane-bound organelles.

**Figure 7:**
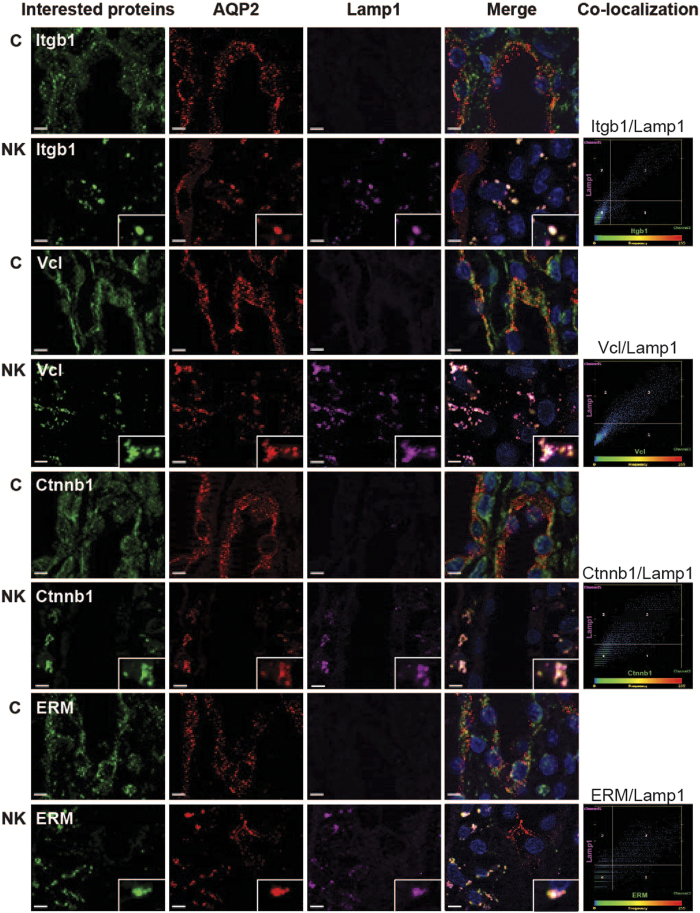
Co-localization of actin cytoskeletal and cell adhesion proteins with autophagy markers. The inner medulla sections of control rats (C) and rats fed with a potassium-free diet for 1 day (NK) were triple-labeled against AQP2 (red), Lamp1 (pink), and down-regulated proteins including Itgb1, Vcl, Ctnnb1, and ERM (green). Insets demonstrate the areas where significant co-localization was observed as a group of puncta, which was not observed in control sections. Scale bar = 4 μm. Scatter plots demonstrate the degree of co-localization between lysosomal marker (Lamp1) and Itgb1 (r^2^ = 0.81, *P* < 0.05), Vcl (r^2^ = 0.54, *P* < 0.05), Ctnnb1 (r^2^ = 0.49, *P* < 0.05), or ERM (r^2^ = 0.42, *P *< 0.05) after potassium deprivation.

Based on the ultrastructural analysis revealing the extensive presence of autophagic structures in IMCD cells of potassium deprived rats, we further investigated the potential role of autophagy in down-regulation of the proteins detected by proteomic analysis. Co-localization studies were performed by immunofluorescence using antibodies against markers specific for autophagosomes (LC3) and lysosomes (Lamp1). First, numerous puncta containing AQP2 were seen in IMCD cells of the potassium-deprived rats, with significant co-localization with both LC3 and Lamp1 (Fig. 6A), indicating that AQP2 was located in autophagolysosomes. Representative scatter plots demonstrate the high degree of co-localization between AQP2 and LC3 or Lamp1 after potassium deprivation (Fig. 6B). A bar graph summarizes Pearson correlation coefficients of AQP2 and LC3 or Lamp1 from 30 co-localized puncta (Fig. 6C). Other down-regulated proteins identified, including Itgb1, Vcl, Ctnnb1, Ezrin-Moesin-Radixin (ERM), Cttn, and RhoA were also co-localized with AQP2 and Lamp1 in a punctate pattern, also indicating their localization in autophagolysosomes (Fig. 7 and Supplementary Fig. 4). Scatter plots demonstrate the degree of co-localization between Itgb1 and Lamp1 (Pearson correlation coefficient*, R* = 0.90), Vcl and Lamp1 (*R* = 0.74), Ctnnb1 and Lamp1 (*R* = 0.70) as well as ERM and Lamp1(*R* = 0.65) in potassium-free condition (Fig. 7). No co-localization of these proteins with LC3/Lamp1 was observed in control rats.

The immunofluorescence results were confirmed by double-label immunogold electron microscopy with AQP2 and LC3 (Fig. 6D), Lamp1 (Supplementary Fig. 5), or another lysosomal marker, cathepsin D (Fig. 6E). AQP2 and LC3 or AQP2 and cathepsin D localize in similar membrane-bound organelles throughout the kidney IMCD cells of potassium deprived rats. Similar structures were not frequently observed in control rats. The presence of AQP2 in autophagosomes and autophagolysosomes in the IMCD of potassium-deprived rats supports the role of autophagic protein degradation as the mechanism responsible for the down-regulation of IMCD proteins in the early stage of hypokalemia-induced NDI.

### Potassium re-feeding ameliorates autophagic degradation of AQP2 induced by potassium deprivation

**Figure 8:**
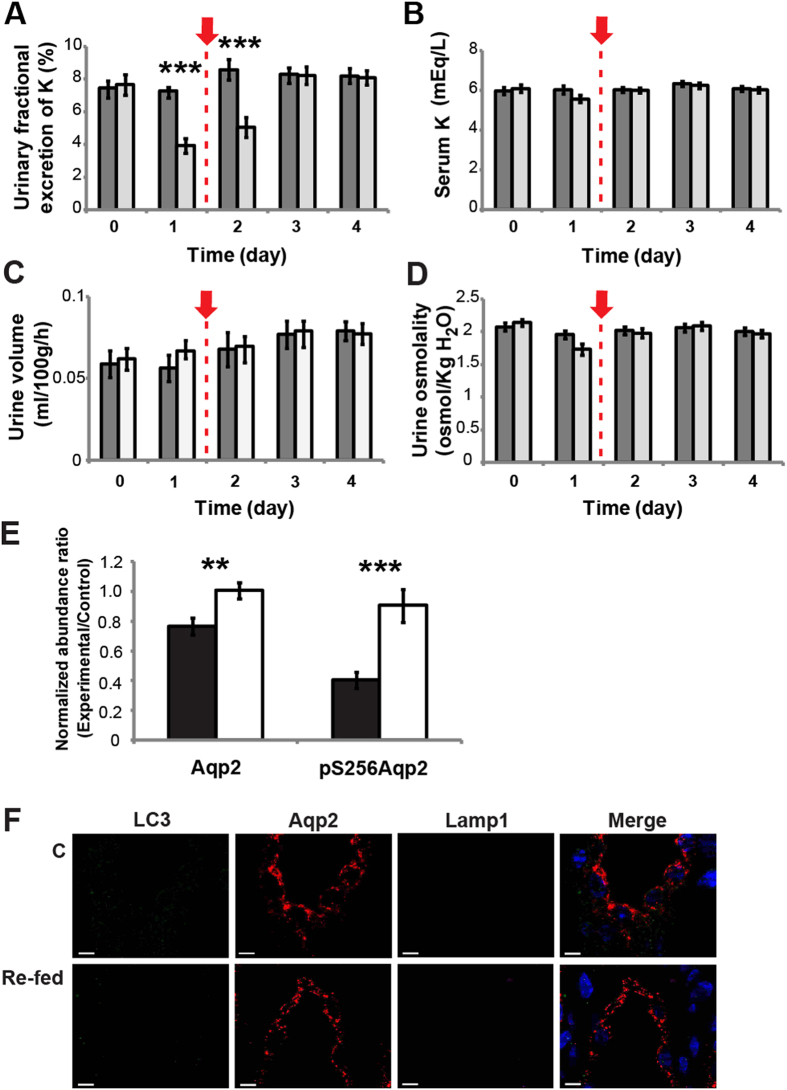
Responses of potassium-deprived rats to potassium re-feeding. Urinary fractional excretion of potassium (**A**), serum potassium (**B**), urine volume (**C**), and urine osmolality (**D**) were measured in rats receiving a normal chow diet (■, n = 7) for 4 days or a potassium-free diet for 1 day followed by a normal chow diet on days 2, 3, and 4 (“re-fed” rats; □, n = 7, red arrows indicate the start of refeeding). The units for urinary fractional excretion of potassium (shown as %) are a ratio of urinary:plasma potassium concentration normalized to that of urinary:plasma creatinine concentration. (**E**) Normalized abundance ratios for AQP2 and pS256AQP2, quantified by immunoblotting of inner medullary proteins from potassium-deprived rats (■, n=7) and re-fed rats (□, n = 7) at day 4. The normalized ratios were obtained by dividing the band intensities from each experimental animal by the average intensity of the control group (control for either potassium-deprived rats or re-fed rats). (**F**) The inner medulla sections of control rats (C) and re-fed rats were probed using antibodies against LC3 (green), Lamp1 (pink), and AQP2 (red).

Potassium replenishment is known to reverse hypokalemia-induced NDI in clinical patients^28^,^29^, and also in experimental animals fed a potassium-deficient diet^12^,^30^. In order to further investigate the role of autophagy induced by potassium deprivation in producing the NDI phenotype, we performed a potassium re-feeding experiment. Following 1 day of potassium deprivation, rats were switched to a standard diet (i.e., containing the normal potassium content) for an additional 3 days. As with the potassium deprivation experiments, the earliest detectable change after potassium re-feeding was in the urinary fractional excretion of potassium, which was rapidly normalized within 24–48 hours (compare Fig. 8A to Fig. 1A). As expected, potassium re-feeding also reversed both the development of hypokalemia and the NDI phenotype, normalizing serum potassium level (Figs 8B vs. 1B), urine volume (Figs 8C vs. 1C) and urine osmolality (Figs 8D vs. 1D). Concomitantly, potassium re-feeding restored the abundances of AQP2 and pS256 AQP2 in the inner medulla to normal levels (Fig. 8E). In addition, the autophagy markers, LC3 and LAMP1, were no longer detectable and/or no longer co-localized with AQP2 after the refeeding (Fig. 8F). Thus, the recovery of the normal phenotype coincided with the restoration of AQP2 levels and the halting of its degradation via autophagy.

## Discussion

Previous studies of hypokalemia-induced NDI in animal models have mostly investigated the long-term effects of potassium deprivation (several days to weeks), focusing on a limited number of target molecules in the kidney such as AQP2, urea transporters, sodium channels, the transcriptional activator TonEBP, and cAMP^11^,^12^,^13^,^14^,^31^,^32^,^33^. However, a study by Amlal *et al.* demonstrated that urinary concentrating defects induced by potassium deprivation can actually be detected much earlier (i.e., within a day)^13^. Thus, in the present study, we devised a strategy to better understand the pathogenesis of this condition by (i) investigating changes that occur in the initial stage of hypokalemia-induced NDI and (ii) applying an unbiased discovery approach (i.e., mass spectrometry-based proteomics). While serum potassium, urine volume, and cAMP levels remained unchanged at day 1, the early occurrence of a urinary concentrating defect (at day 1) was apparent in our study (Supplementary Fig. 1); in addition, early reduction in AQP2 protein abundance and phosphorylation and urinary potassium excretion (at day 1) were also detectable.

The reason that these early changes could occur in the absence of a decreased serum potassium level is the function of a feedforward regulatory system, originating with the gut dietary potassium sensor^19^,^20^,^21^. Although some major elements of the entire system are not yet completely understood, potassium sensing cells in the gut splanchnic bed (especially ones that supply stomach^19^ and hepato-portal vein^34^) transmit potassium intake signals to the brain, probably the hypothalamus, which subsequently relays signals to the pituitary. Hypophysectomy ameliorates the feedforward regulation of the kidney in response to changes in dietary potassium level, but the pituitary peptide(s) responsible for this regulation has not been identified^21^,^35^. As a result of this neuroendocrine feedforward system, a rapid response to decreased dietary potassium intake occurs^21^, producing an early decrease in urinary potassium excretion (significant on day 1, Fig 1A), thereby conserving body potassium^20^,^30^.

An early reduction in AQP2 protein was also previously demonstrated by Amlal *et al.*^13^, occurring along the entire collecting duct system as early as the first 24–48 hours after starting a potassium-deficient diet, however, the mechanism for AQP2 down-regulation remained unknown. The present study is the first to demonstrate that AQP2 in IMCD cells is sequestered in autophagosomes/autolysosomes as an early event following potassium deprivation. Since down-regulation of AQP2 is sufficient to induce a urinary concentrating defect^36^,^37^, it is at least plausible, if not highly probable, that autophagic degradation is the mechanism for down-regulation of AQP2 in hypokalmemia-induced NDI. Furthermore, this finding of early hypokalemia-induced autophagy in the IMCD is supported by a previous study showing that lysosomal enzyme activities in the renal inner medulla increased after 1 day of potassium depletion^38^.

Autophagy is a highly conserved and regulated catabolic process that involves the sequestration and transport of macromolecules and organelles to the lysosome for degradation^39^. In response to cellular stress, the basal activity of this normal process is increased to maintain cellular homeostasis. Autophagy is not simply a bulk degradation process but it can also be highly selective^40^,^41^,^42^,^43^, and in fact, we observed no change in abundance for a majority (>70%) of the proteins identified in the proteomic analysis. In addition to AQP2, 127 other proteins, including at least three groups of functionally-related proteins, appeared to be selectively down-regulated at this very early time point, prior to the appearance of NDI features. For example, proteomic analysis revealed down-regulation of 41 mitochondrial proteins, including a group of 19 proteins involved in energy production, which was also supported by the evidence of mitochondrial damage and mitophagy from our EM analysis. These findings are consistent with the study by Aithal *et al.* which demonstrated defective mitochondrial energy production as well as depletion of the mitochondrial content of potassium, magnesium and calcium in the renal medulla of rats fed with a potassium-deficient diet for 5 weeks^44^.

The other groups of down-regulated proteins identified by proteomic analysis were involved in actin cytoskeleton organization and cell-cell adhesion (Table 1). The co-localization with autophagy markers for six proteins representing these two groups indicated that autophagy also appears to play a role in selective down-regulation of these groups of proteins. These findings were also supported by histopathology and EM analysis showing disruption of cell-cell junctions between IMCD cells and disorganized actin filaments at tight junctions. Similarly, a previous *in vitro* study demonstrated that potassium depletion inhibited formation of tight junctions, desmosomes and bundled stress fibers in Madin Darby canine kidney (MDCK) epithelial cells^45^.

One question that arises from the results of this study is how potassium deprivation is linked to mitochondrial dysfunction and the other observed changes in cellular structures. It is well established that the inner mitochondrial membrane contains at least four different potassium channels and at least one K^+^/H^+^ antiporter^46^,^47^. These channels function in a “K^+^ cycle” to help regulate changes in mitochondrial matrix volume, membrane potential and rate of ATP synthesis, as well as to modulate the generation of reactive oxygen species (ROS) by mitochondria. In a previous study, intracellular potassium ([K^+^]_i_) in inner medullary cells was significantly decreased between 24 and 72 hours after initiating a low-potassium diet^30^. Thus, while we did not measure [K^+^]_i_ in IMCD cells of our rats fed with a potassium-free diet (*vs.* a low-potassium diet in the aforementioned study), it is likely that [K^+^]_i_ was decreased at the 24 hour time-point used in the present study. Based on these factors, we postulate that decreased [K^+^]_i_ should alter mitochondrial potassium channel activities and potassium balance across the inner mitochondrial membrane. Altered mitochondrial potassium balance would then lead to perturbations in mitochondrial volume, membrane potential, and rates of ATP and ROS synthesis by mitochondria. Indeed, mitochondrial volume changes (i.e., swelling) were observed in our study (Fig. 5F). Thus, it is plausible that mitochondrial damage (from ROS and any of the other mitochondrial perturbations mentioned above) was induced at the early stage of potassium deprivation. This damage would initiate mitophagy and subsequent cellular ATP depletion followed by the downstream activation of autophagic flux, resulting in the structural defects and other changes that we observed. Consistent with this hypothesis, disruption of adherens junctions has been observed in various ATP-depletion models *in vitro* (cultured MDCK cells)^48^. In addition, autophagy has been reported in another ATP-depletion model, i.e., an ischemia model in the human renal proximal tubular cell line, HK-2^49^.

Our data indicate that potassium deprivation lead to autophagic degradation of a specific subset of cellular proteins, including AQP2 and the other proteins mentioned above; from this study, it is not possible to determine whether the down-regulation of the latter proteins is linked in a direct or causal way to AQP2 degradation or the NDI phenotype. Although our results clearly showed that activation of autophagy (and mitophagy) in IMCD cells was an early event in response to potassium depletion, we have not definitively demonstrated that autophagic degradation is responsible for the NDI phenotype. In order to formally demonstrate that autophagy produces NDI, we would need to show that inhibition of autophagy prevents the urinary concentrating defect and other manifestations of NDI. Unfortunately, the inhibitors that are commonly used to block autophagy (chloroquine, 3-MA, etc.) are non-specific, only act at late stage in the autophagy process, and/or cannot be used for *in vivo* studies (i.e., animal studies)^50^,^51^,^52^,^53^,^54^, and results using these inhibitors are often quite variable both *in vivo* and *in vitro*^55^,^56^. Furthermore, no *in vitro* model exists that recapitulates the hypokalemia-induced NDI phenotype. The only other way to inhibit autophagy is to knockout various autophagy-related genes, which is not feasible using a rat model. One practical, although somewhat less direct, approach performed in this study was a re-feeding experiment. The findings demonstrated that the recovery of the normal phenotype after potassium replenishment coincided with the normalization of AQP2 levels and its co-localization with autophagy markers was abolished, indicating that autophagic degradation, at least for AQP2, was arrested. Nevertheless, definitive demonstration that autophagy is the mechanism responsible for hypokalemia-induced NDI will require a strategy which specifically blocks the early stage of autophagy and is suitable for *in vivo* use.

## Methods

Brief descriptions of key experimental procedures are provided below. For complete details see supplementary material online.

### Experimental Design and Statistical Rationale

#### Experimental animals

All experiments were approved by the Animal Care and Use Committee of the Faculty of Medicine, Thammasat University and were carried out in “accordance” with the approved guidelines. Experiments were conducted on male Sprague-Dawley rats weighing 180–200 g. Rats were randomly assigned into 2 groups: rats fed with a potassium-free diet (TD.88239, Harlan, WI, USA) or control rats fed with standard rat chow.

#### Sample preparation for proteomic analysis

IMCD tissue was prepared from renal inner medullas according to Stokes *et al.*^57^ with modifications^58^. Three independent pairs of samples were prepared using four potassium-deprived rats versus four control rats for each pair (IMCD tissue in each group was pooled prior to homogenization). Each pair of samples (100 μg protein per sample) was subjected to 1D SDS-PAGE and in-gel trypsinization was performed as previously described^59^.

#### LC-MS/MS analysis

Samples were analyzed using an LTQ Orbitrap XL mass spectrometer (Thermo Scientific, San Jose, CA). Mass spectra were searched using two different search algorithms, SEQUEST (Bioworks version 3.31 SP1)^60^ and InsPecT (version 20070905)^61^, with the rat RefSeq database (National Center for Biotechnology Information, March, 2010, 30734 entries) which also included a list of common contaminating proteins from other species (e.g., porcine trypsin). Precursor ion tolerance was 10 ppm, while fragment ion tolerance was 0.8 Da. Three missed trypsin cleavage sites were allowed. Static modifications included carbamidomethylation of cysteine (+57.021 Da). Variable modifications included oxidation of methionine (+15.995 Da). All datasets were filtered to achieve a false discovery rate of <1%, estimated based on target-decoy analysis^62^ based on an absolute Xcorr threshold of 0.4. The InsPecT search results were filtered to a random match probability <1%.

#### Label-free quantification

Peptide quantification by a label-free technique was performed using QUOIL software^24^. Only proteins with peptides quantified in all three biological replicates were used for statistical significance testing. The log_2_ of NK/Control protein abundance ratio was used as the basis for calculation of the average abundance change and statistical significance by unpaired t-test for each protein.

#### Bioinformatics

The DAVID bioinformatics resource (Database for Annotation, Visualization and Integrated Discovery, NIAID, http://david.abcc.ncifcrf.gov/)^26^ was used to extract the list of significantly enriched functional annotation clusters associated with the proteins exhibiting significantly altered abundance as a result of potassium deprivation.

#### Targeted LC-MS/MS

For targeted LC-MS/MS experiments, new IMCD samples were prepared as described above, except that one pair of samples (four potassium-deprived rats versus four control rats for each pair) was analyzed. Peptides from selected proteins (identified as down-regulated in the initial proteomic analysis) were chosen based on the following criteria: uniqueness to the targeted proteins; fully tryptic; and absence of potentially modified amino acids. Skyline^63^ was used to create an inclusion list of peptide m/z ratios for targeted proteomics experiments. Targeted LC-MS/MS analysis was performed using a Q-Exactive Orbitrap mass spectrometer (Thermo Scientific, San Jose, CA) (see Full Methods in Supplementary Information). Peak areas for each peptide were extracted using Skyline software^63^.

#### Quantitative Real-Time PCR

The experiment was performed as previously described^64^. The rat primers are provided in Supplemental methods. All values were normalized to *18s rRNA*.

#### Immunoblotting

Immunoblotting was carried out as previously described^65^. A list of antibodies used can be found in Supplemental methods. To normalize samples for gel loading, samples were first analyzed on a separate gel, stained with Coomassie blue, scanned, and quantified for final justification of protein concentration in each sample prior to immunoblotting experiments^66^.

#### Electron microscopy

Fresh renal inner medullas were prepared as described in Supplemental methods. The ultrathin sections were examined under a transmission electron microscope (FEI Tecnai G2 TWIN 200 kV).

#### Immunofluorescence

Immunofluorescence labeling of kidney sections was done as previously described^67^. A list of antibodies used can be found in Supplemental methods. Fluorescence images were acquired using an Axio Observer Z1 microscope in conjunction with ApoTome2 (Carl Zeiss, Jena, Germany).

#### Immunogold electron microscopy

The procedures have been described previously^68^. A list of antibodies used can be found in Supplemental methods. The sections were examined in a FEI Morgagni electron microscope.

#### Re-feeding experiment

An experiment was conducted on male Sprague-Dawley rats weighing 180–200 g. Fourteen rats were randomly assigned into 2 groups: 1) seven rats fed with a potassium-free diet for 1 day followed by standard rat chow for 3 days; and 2) seven rats fed with standard rat chow for 4 days. Urine and serum parameters were measured at study onset and daily. Kidneys were harvested and inner medullas were isolated, processed, and analyzed as described above.

## Additional Information

**How to cite this article**: Khositseth, S. *et al.* Autophagic degradation of aquaporin-2 is an early event in hypokalemia-induced nephrogenic diabetes insipidus. *Sci. Rep.* **5**, 18311; doi: 10.1038/srep18311 (2015).

## Supplementary Material

Supplementary Information

Supplementary Data 1

Supplementary Data 2

## Figures and Tables

**Table 1:** A list of cell-cell adhesion and cytoskeleton related proteins which correlate with proteomic analysis and kidney histopathology.

Functions	Proteins
Cadherin-based adherens	β-catenin (Cttnb1)*, vinculin (Vcl)*, cadherin-16 (Cdh16), α-actinin-4 (Actn4)
Focal adhesion protein	integrin β-1 (Itgb1)*, vinculin (Vcl)*, filamin-B (Flnb), α-actinin-4 (Actn4)
Actin cytoskeleton organization	ezrin (Ezr)*, transforming protein RhoA precursor (RhoA)*, integrin β-1 (Itgb1)*, calreticulin precursor (Calr), EH domain-containing protein 2 (Ehd2), keratin, type I cytoskeletal 19 (Krt19), spectrin alpha chain, non-erythrocytic 1 (Sptan1), ras-related protein Ral-A (Rala)

*Proteins co-localized with AQP2 and Lamp1 in IMCD of potassium-deprived rats as demonstrated by immunofluorescence in this study.
