# Supplementary material for: Autophagic degradation of aquaporin-2 is an early event in hypokalemia-induced nephrogenic diabetes insipidus
Source: Sci Rep. 2015 Dec 17;5:18311. doi: 10.1038/srep18311 (PMC4682130; doi:10.1038/srep18311)
Supplement: Supplementary Information [file srep18311-s1.pdf]

Supplementary information

Autophagic degradation of aquaporin-2 is an early event in hypokalemia-induced nephrogenic diabetes insipidus

Sookkasem Khositseth[1*], Panapat Uawithya[2], Poorichaya Somparn[3], Komgrid Charngkaew[4], Nattakan Thippamom[1], Jason D. Hoffert[5], Fahad Saeed[6], D. Michael Payne[3], Shu Hui Chen[7], Robert A. Fenton[8] and Trairak Pisitkun[3,8,9*]

[1]*Department of Pediatrics, Faculty of Medicine, Thammasat University,*[2]*Department of Physiology, Faculty of Medicine Siriraj Hospital, Mahidol University,* [3]*Systems Biology Center, Research Affairs, Faculty of Medicine, Chulalongkorn University,* [4]*Department of Pathology, Faculty of Medicine Siriraj Hospital, Mahidol University,* [5]*National Institute of Diabetes and Digestive and Kidney, Bethesda, MD,*[6]*Department of Electrical & Computer Engineering and Department of Computer Science, Western Michigan University,* [7]*Department of Chemistry, National Cheng Kung University, Taiwan ,*[8]*Department of Biomedicine and Center for Interactions of Proteins in Epithelial Transport, Aarhus University, Denmark,* [9]*Epithelial Systems Biology Laboratory, National Heart, Lung, and Blood Institute, Bethesda, MD*

FULL METHODS

Experimental animals. All experiments were approved by the Animal Care and Use Committee of the Faculty of Medicine, Thammasat University. Experiments were conducted on male Sprague-Dawley rats weighing 180-200 g. After 3 days of acclimatization in metabolic cages, rats were randomly assigned into 2 groups: rats fed with a potassium-free diet (TD.88239, Harlan, WI, USA) or control rats fed with standard rat chow. Rats were offered the same amount of food and free access to water. In order to identify the onset of NDI in our setting, rats were monitored for 3 days. Body weight, water intake as well as urine and serum (blood drawn from tail vein) parameters were measured at study onset and daily.

Sample preparation for proteomic analysis. IMCD was prepared from renal inner medullas according to Stokes et al.[1] with modifications[2]. IMCD tubules were lysed in 150 μl of lysis buffer (1.5% SDS, 10 mM Tris, pH 6.8) containing protease inhibitors. Protein samples were sonicated and centrifuged at 14,000 *g* for 10 min at 4°C and the supernatants were used for proteomic analysis. Three independent pairs of samples were prepared using four potassium-deprived rats versus four control rats for each pair (IMCD proteins in each group were pooled). Each pair of samples (100 μg per sample) was subjected to one-dimensional SDS-PAGE. The gel was stained with colloidal Coomassie blue, destained, and sliced into 5 blocks per lane (identical slicing on both lanes was performed for each pair). Each block was minced into small pieces (1–1.5 mm^3) and in-gel trypsinization was performed as previously described[3]. Tryptic peptides were extracted with 50% acetonitrile (ACN)/0.1% formic acid (FA), desalted, dried under vacuum, and dissolved in 0.1% FA for subsequent LC-MS/MS analysis.

LC-MS/MS analysis and database searching. Samples were analyzed using an LTQ Orbitrap XL mass spectrometer (Thermo Scientific, San Jose, CA) equipped with a nanoflow

Ultra-high Pressure Liquid Chromatography (nanoACQUITY UPLC, Waters Corp., MA). Mass spectra were searched using two different search algorithms, SEQUEST (Bioworks version 3.31 SP1)[4] and InsPecT (version *20070905*)[5], on rat RefSeq database (National Center for Biotechnology Information, March, 2010, 30734 entries) which included a list of common contaminating proteins from other species. Precursor ion tolerance was 10 ppm, while fragment ion tolerance was 0.8 Da. Three missed trypsin cleavage sites were allowed. Static modification included carbamidomethylation of cysteine (+57.021 Da). Variable modification included oxidation of methionine (+15.995 Da). All datasets were filtered to achieve a false discovery rate of < 1%, estimated based on target-decoy analysis[6]. Peptides matching to multiple protein isoforms were identified using ProMatch software[7].

Label-free quantification. Peptide quantification by a label-free technique was performed using QUOIL software[8]. This program calculates relative peptide abundance based on the area under the extracted ion chromatogram of all peptides in both potassium-free (NK) and control conditions. The median peptide abundance of all peptides identified for a given protein was used to infer the protein abundance change. Only proteins with peptides quantified in all three biological replicates were used for statistical significance testing. The $\log_2$ of NK/Control protein abundance ratio was used as the basis for calculation of the average abundance change and statistical significance by unpaired t-test for each protein.

Bioinformatics. Automated Bioinformatics Extractor, ABE (http://helixweb.nih.gov/ESBL/ABE/), was used to extract protein names, gene symbols, accession numbers, and Gene Ontology (GO) terms for all data. The DAVID bioinformatics resources (Database for Annotation, Visualization and Integrated Discovery, NIAID, http://david.abcc.ncifcrf.gov/)[9] was used to extract the list of significantly enriched functional

annotation clusters, KEGG pathways, and biological process and cellular component GO terms associated with the significantly changed proteins induced by potassium deprivation.

Targeted LC-MS/MS. Peptides from candidate proteins were selected based on prior non-targeted LC-MS/MS data. Skyline software[10] was used to create an inclusion list of all selected peptides including three isotopes per peptide. Targeted LC-MS/MS analysis was performed using a Q-Exactive Orbitrap mass spectrometer (Thermo Scientific, San Jose, CA). Peak areas for each peptide were extracted and integrated using Skyline software.

LC-MS/MS analysis was performed using a standard top 15 method on Q-Exactive orbitrap MS (Thermo Scientific) in conjunction with a Proxeon Easy-nLC II HPLC (Thermo Scientific) and Proxeon nanospray source. The digested peptides were reconstituted in 2% acetonitrile /0.1% trifluoroacetic acid and loaded onto a 100 micron x 25 mm Magic C18 100Å 5U trap reverse phase trap where they were desalted online before being separated using a 75 micron x 150 mm Magic C18 200Å 3U reverse phase column. Data dependent MS/MS data was collected using higher energy collision dissociation (HCD). Peptides were eluted using a gradient of 0.1% formic acid (A) and 100% acetonitrile (B). A 90 minute gradient was ran with 5% to 35% B over 75 minutes, 35% to 80% B over 5 minutes, 80% B for 1 minute, 80% to 5% B over 1 minute, and finally held at 5% B for 8 minutes. The mass spectrometer was operated with a spray voltage of 1.8kV, a capillary temperature of 275°C, and a normalized collision of 27%.

Quantitative Real-Time PCR. Total RNA was extracted from whole inner medullas of 7 control and 7 potassium-depleted rats using the RNeasy Mini Kit (Qiagen, Venlo, Netherlands) following the manufacturer's protocol. 0.5 µg total RNA were used for first strand cDNA synthesis using an oligo(dT) primer. PCR was performed using 0.5 µl of the first strand cDNA

mixture using gene specific primers (see below). PCR was performed with the following conditions: an initial denature at 95°C for 2 min, followed by heating at 95°C for 10 s and 60°C for 10 s repeated for 40 cycles.

Real-time RT-PCR was carried out in a 20 µl reaction volume composed of 2 µl of the first strand cDNA and 18 µl of a real-time PCR master mix containing 10 µl of 2X Power SYBR Green PCR Master Mix (Applied Biosystem, Calsbad, USA), 0.5 µM forward primer, and 0.5 µM reverse primer, using the 7500 Fast System (Applied Biosystems, Calsbad, USA). The PCR amplicon for *18s* rRNA was used for developing standard curves. Quantification between groups was performed using the comparative Ct method.

Gene name	Primers
Aqp2	Forward 5'-GAC TGT GCT TAG TGC ATC TCA TTT TAT-3'<br>Reverse 5'-AGG ATC ATG CAA ACT CTG TTC GT-3'
Atp5b	Forward 5'-CTA TGA CCA TCT CCC GGA ACA-3'<br>Reverse 5'-CAG CTT GTC AGC CTT TGC C-3'
Itgb1	Forward 5'-CGA TAG GTC CAA CGG CTT AAT CTG TG-3'<br>Reverse 5'-CTT GGA ACT TGG GAT CTG TGC ACT-3'
Vcl	Forward 5'-TCA GAT GAG GTG ACT CGG TTG G-3'<br>Reverse 5'-GGGTGCTTATGGTTGGGATTCG-3'
Mdh1	Forward 5'-ATC TCA AAT TGC TCT TAA ACT CG-3'<br>Reverse 5'-TGC TGC ACA GTC GTG ATG AA-3'
18s	Forward 5'-GTA ACC CGT TGA ACC CCA TT-3'<br>Reverse 5'-CCA TCC AAT CGG TAG TAG CG-3'
Cdh16	Forward 5'-TCC CGA TGG AAG CAC CAC C-3'<br>Reverse 5'-CCC AGT GCA CCT GGG CAA T-3'

Immunoblotting. Polyclonal antibodies against Aqp2[11], pS256-Aqp2[12], pS261-Aqp2[13], and UT-A1/3[14] were kindly provided by Dr. Mark A. Knepper (NIH). Polyclonal antibodies against Ctnnb1 (PA5-17784) and Cttn (PA5-17730) were purchased from Pierce (Rockford, IL). Monoclonal antibody against RhoA (2117) and polyclonal antibody against Caspase-3 (9662) were purchased from Cell Signaling Technology (Danvers, MA). Immunoblotting was carried

out as previously described[15]. To normalize samples for gel loading, samples were first analyzed on a separate gel, stained with Coomassie blue, scanned, and quantified for final justification of protein concentration in each sample prior to immunoblotting experiments[16].

cAMP measurement. Intracellular cAMP levels in IMCD were measured by a Direct cAMP ELISA kit (ADI-900-066, Enzo Life Sciences, Farmingdale, NY).

Electron microscopy. Fresh renal inner medullas from control and potassium-deprived groups were dissected, minced into 1 mm^3 cubes, and fixed in 4% paraformaldehyde with 1% glutaraldehyde 12 hours fixative. Post-fixation was performed in 1% osmium tetroxide in Millonig buffer for 1 h at 4 °C followed by dehydration in a graded ethanol series and propylene oxide. The tissues were embedded in epoxy resin and polymerized at 60 °C for 48 hours. Ultrathin sections were cut and mounted on naked copper grids before staining with 2% uranyl acetate and lead citrate solutions then examined under a transmission electron microscope (FEI Tecnai G2 TWIN 200 kV).

Immunofluorescence. Immunofluorescence labeling of kidney sections was done as previously described[17]. Primary antibodies used were against Aqp2 (LL265)[18]; Lamp1 (17768) and LC3 (282666) from Santa Cruz (CA, USA); Itgb1 (4706), Vcl (4650), RhoA (3142), and ERM (3142) from Cell Signaling Technology (Danvers, MA); Ctnnb1 (PA5-17784) and Cttn (PA5-17730) from Pierce (Rockford, IL); and UT-A1 (L179)[14]. Secondary antibodies were conjugated with Alexa Fluor 488 (anti-rabbit: green channel), 555 (anti-chicken: red channel), and 633 (anti-mouse: pink channel) (Invitrogen, CA, USA). Nuclei were stained with 4', 6-diamidino-2-phenylindole (DAPI, D3571, Invitrogen) and F-actin with rhodamine phalloidin (R415, Life Technologies, Grand Island, NY). Fluorescence images were acquired using an Axio Observer Z1 microscope in conjunction with ApoTome2 (Carl Zeiss, Jena, Germany).

Immunogold electron microscopy. All procedures have been described in detail previously[19]. In brief, after anaesthesia, rat kidneys were removed, inner medullas dissected out, fixed in 4% paraformaldehyde solution in 0.1 M Na^+cacodylate, pH 7.2, for 3 h, infiltrated with 2.3 M sucrose, and frozen in liquid nitrogen. Ultrathin (70 nm) cryosections were cut on a Reichert Ultracut S cryo-ultramicrotome (Leica) and mounted. Sections were blocked by incubation in PBS containing 0.05 M glycine and 1% BSA before incubation overnight at 4°C with various antibodies diluted in PBS containing 0.1% skim milk powder. Primary antibodies used were a rabbit polyclonal antibody against AQP2[19], and goat polyclonal antibodies against Cathepsin D (Research Diagnostics), MAP LC3β (clone G-2, Santa Cruz) or Lamp1 (C20, Santa Cruz). Primary antibodies were visualized using various combinations (see figure legends) and sequential application of donkey anti-rabbit IgG, goat anti-rabbit IgG or donkey anti-goat IgG conjugated to 5nm, 6nm or 10nm colloidal gold particles (BioCell Research Laboratories) diluted 1:50 in PBS with 0.1% skim milk powder and polyethylene glycol (5 mg/ml). After counterstaining with 0.3% uranyl acetate in 1.8% methylcellulose for 10 min, sections were examined in a FEI Morgagni electron microscope. Immunolabeling controls included incubation without primary antibodies, use of only one secondary antibody (in double labeling experiments) and use of alternatively sized colloidal gold conjugated secondary antibodies.

Re-feeding experiment. An experiment was conducted on male Sprague-Dawley rats weighing 180-200 g. After 3 days of acclimatization in metabolic cages, fourteen rats were randomly assigned into 2 groups: 1) seven rats fed with a potassium-free diet (TD.88239, Harlan, WI, USA) for 1 day followed by standard rat chow for 3 days; 2) seven control rats fed with standard rat chow for 4 days. Body weight, water intake as well as urine and serum (blood drawn

from tail vein) parameters were measured at study onset and daily. Kidneys were harvested and inner medullas were isolated, processed, and analyzed as described above.

References

1 Stokes, J. B., Grupp, C. & Kinne, R. K. Purification of rat papillary collecting duct cells: functional and metabolic assessment. *The American journal of physiology* **253**, F251-262 (1987).

2 Pisitkun, T. *et al.* High-throughput identification of IMCD proteins using LC-MS/MS. *PhysiolGenomics* **25**, 263-276, doi:00214.2005 [pii]10.1152/physiolgenomics.00214.2005 (2006).

3 Pisitkun, T., Shen, R. F. & Knepper, M. A. Identification and proteomic profiling of exosomes in human urine. *Proc Natl Acad Sci U S A* **101**, 13368-13373, doi:10.1073/pnas.04034531010403453101 [pii] (2004).

4 Yates, J. R., 3rd, Eng, J. K., McCormack, A. L. & Schieltz, D. Method to correlate tandem mass spectra of modified peptides to amino acid sequences in the protein database. *Anal Chem* **67**, 1426-1436 (1995).

5 Tanner, S. *et al.* InsPecT: identification of posttranslationally modified peptides from tandem mass spectra. *Anal Chem* **77**, 4626-4639, doi:10.1021/ac050102d (2005).

6 Elias, J. E. & Gygi, S. P. Target-decoy search strategy for increased confidence in large-scale protein identifications by mass spectrometry. *Nat Methods* **4**, 207-214, doi:nmeth1019 [pii]10.1038/nmeth1019 (2007).

7 Tchapyjnikov, D. *et al.* Proteomic profiling of nuclei from native renal inner medullary collecting duct cells using LC-MS/MS. *Physiol Genomics* **40**, 167-183, doi:00148.2009 [pii]10.1152/physiolgenomics.00148.2009.

8 Wang, G., Wu, W. W., Zeng, W., Chou, C. L. & Shen, R. F. Label-free protein quantification using LC-coupled ion trap or FT mass spectrometry: Reproducibility, linearity, and application with complex proteomes. *J Proteome Res* **5**, 1214-1223, doi:10.1021/pr050406g (2006).

9 Dennis, G., Jr. *et al.* DAVID: Database for Annotation, Visualization, and Integrated Discovery. *Genome Biol* **4**, P3 (2003).

10 MacLean, B. *et al.* Skyline: an open source document editor for creating and analyzing targeted proteomics experiments. *Bioinformatics* **26**, 966-968, doi:btq054 [pii]10.1093/bioinformatics/btq054 (2010).

11 Hoffert, J. D. *et al.* Vasopressin-stimulated increase in phosphorylation at Ser269 potentiates plasma membrane retention of aquaporin-2. *J Biol Chem* **283**, 24617-24627, doi:M803074200 [pii]10.1074/jbc.M803074200 (2008).

12 Nishimoto, G. *et al.* Arginine vasopressin stimulates phosphorylation of aquaporin-2 in rat renal tissue. *The American journal of physiology* **276**, F254-259 (1999).

13 Hoffert, J. D. *et al.* Dynamics of aquaporin-2 serine-261 phosphorylation in response to short-term vasopressin treatment in collecting duct. *American journal of physiology. Renal physiology* **292**, F691-700, doi:10.1152/ajprenal.00284.2006 (2007).

14 Nielsen, S. *et al.* Cellular and subcellular localization of the vasopressin- regulated urea transporter in rat kidney. *Proc Natl Acad Sci U S A* **93**, 5495-5500 (1996).

15 Pisitkun, T. *et al.* Akt and ERK1/2 pathways are components of the vasopressin signaling network in rat native IMCD. *Am J Physiol Renal Physiol* **295**, F1030-1043, doi:90339.2008 [pii]10.1152/ajprenal.90339.2008 (2008).

16 Chou, C. L., Ma, T., Yang, B., Knepper, M. A. & Verkman, A. S. Fourfold reduction of water permeability in inner medullary collecting duct of aquaporin-4 knockout mice. *The American journal of physiology* **274**, C549-554 (1998).

17 Yu, M. J., Pisitkun, T., Wang, G., Shen, R. F. & Knepper, M. A. LC-MS/MS analysis of apical and basolateral plasma membranes of rat renal collecting duct cells. *Mol.Cell Proteomics.* **5**, 2131-2145 (2006).

18 Barile, M. *et al.* Large scale protein identification in intracellular aquaporin-2 vesicles from renal inner medullary collecting duct. *Mol.Cell Proteomics.* **4**, 1095-1106 (2005).

19 Fenton, R. A. *et al.* Acute regulation of aquaporin-2 phosphorylation at Ser-264 by vasopressin. *Proc Natl Acad Sci U S A* **105**, 3134-3139, doi:0712338105 [pii]10.1073/pnas.0712338105 (2008).

Supplemental Table 1. A list of the significantly decreased proteins in response to potassium depletion (P<0.05 by two tailed t-test and average $\log_2$(NK/Control) ≤ -0.58).

RefSeq	Gene Symbol	Protein Name	$\log_2$ (NK/Control) Mean	SE
NP_058889	Aadat	kynurenine/alpha-aminoadipate aminotransferase, mitochondrial	-4.81	1.45
NP_001094010	Iah1	isoamyl acetate-hydrolyzing esterase 1 homolog	-2.55	0.75
NP_001101788	Atp6v1a	V-type proton ATPase catalytic subunit A	-2.52	0.62
AP_004895	COX2	cytochrome c oxidase subunit II (mitochondrion)	-2.32	0.42
NP_476473	Rhoa	transforming protein RhoA precursor	-2.30	0.78
NP_001005875	Psmd12	26S proteasome non-ATPase regulatory subunit 12	-2.25	0.50
NP_001011915	Fermt2	fermitin family homolog 2	-2.07	0.69
XP_002726232	Slc25a12	LOW QUALITY PROTEIN: calcium-binding mitochondrial carrier protein Aralar1	-1.98	0.62
NP_036732	Me1	NADP-dependent malic enzyme	-1.91	0.45
NP_058977	Psma4	proteasome subunit alpha type-4	-1.70	0.29
NP_877966	Cct4	T-complex protein 1 subunit delta	-1.67	0.43
NP_037042	Arrb1	beta-arrestin-1	-1.64	0.42
NP_037322	Pfkl	6-phosphofructokinase, liver type	-1.56	0.50
NP_114020	Lgals3	galectin-3	-1.54	0.13
NP_112330	Actr3	actin-related protein 3	-1.52	0.11
NP_062230	Ezr	ezrin	-1.46	0.35
NP_001007005	Tuba4a	tubulin alpha-4A chain	-1.41	0.45
NP_058759	Kpnb1	importin subunit beta-1	-1.39	0.17
NP_112262	Akr1a1	alcohol dehydrogenase [NADP(+)]	-1.37	0.17
NP_001101959	Iqgap1	ras GTPase-activating-like protein IQGAP1	-1.37	0.46
NP_058814	Capns1	calpain small subunit 1	-1.26	0.33
NP_445809	Ctnnb1	catenin beta-1	-1.21	0.09
NP_112646	Aifm1	apoptosis-inducing factor 1, mitochondrial precursor	-1.18	0.22
NP_001128170	Aldh4a1	delta-1-pyrroline-5-carboxylate dehydrogenase, mitochondrial	-1.18	0.33
NP_001102031	Ahcyl1	adenosylhomocysteinase-like 1	-1.15	0.24
NP_058895	Ssr4	translocon-associated protein subunit delta precursor	-1.14	0.39
NP_001100073	Cct7	T-complex protein 1 subunit eta	-1.14	0.17
NP_001102025	Lrba	lipopolysaccharide-responsive and beige-like anchor protein	-1.14	0.32
NP_445742	Pgam1	phosphoglycerate mutase 1	-1.11	0.34
NP_446006	Picalm	phosphatidylinositol-binding clathrin assembly protein	-1.10	0.36
NP_062250	Ywhab	14-3-3 protein beta/alpha	-1.10	0.22
NP_036663	Comt	catechol O-methyltransferase	-1.08	0.36
NP_001040552	Eif3a	eukaryotic translation initiation factor 3 subunit A	-1.07	0.16
NP_058726	Pccb	propionyl-CoA carboxylase beta chain, mitochondrial precursor	-1.07	0.18
NP_001008888	Uqcrfs1	cytochrome b-c1 complex subunit Rieske, mitochondrial	-1.06	0.35

Supplemental Table 1.
continued

RefSeq	Gene Symbol	Protein Name	Log2 (NK/Control) Mean	SE
NP_001009641	Pepd	xaa-Pro dipeptidase	-1.06	0.20
NP_112411	Psmc5	26S protease regulatory subunit 8	-1.04	0.26
NP_001100758	Flnb	filamin-B	-1.03	0.17
NP_001011918	Anxa11	annexin A11	-1.03	0.21
NP_942026	Chdh	choline dehydrogenase, mitochondrial	-1.02	0.17
NP_001020050	Dnpep	aspartyl aminopeptidase	-1.02	0.09
NP_001020068	Ehd2	EH domain-containing protein 2	-1.01	0.30
NP_058941	Eef2	elongation factor 2	-1.01	0.14
NP_001013104	Capg	macrophage-capping protein	-1.00	0.20
NP_599191	Atp5b	ATP synthase subunit beta, mitochondrial precursor	-0.99	0.30
NP_001099188	Vps35	maternal embryonic message 3	-0.99	0.33
NP_001004443	Hexa	beta-hexosaminidase subunit alpha precursor	-0.99	0.18
NP_536729	Atp5i	ATP synthase subunit e, mitochondrial	-0.98	0.14
NP_001029298	Nit2	omega-amidase NIT2	-0.97	0.25
NP_001008345	Etf1	eukaryotic peptide chain release factor subunit 1	-0.97	0.11
NP_705893	Hspa4	heat shock 70 kDa protein 4	-0.96	0.15
NP_741984	Sptan1	spectrin alpha chain, non-erythrocytic 1	-0.96	0.16
NP_075211	Tpi1	triosephosphate isomerase	-0.95	0.28
NP_476550	Kif5b	kinesin-1 heavy chain	-0.95	0.29
NP_001041327	Ndufa8	NADH dehydrogenase [ubiquinone] 1 alpha subcomplex subunit 8	-0.94	0.30
NP_446316	Vcp	transitional endoplasmic reticulum ATPase	-0.94	0.18
NP_001026825	Manba	beta-mannosidase precursor	-0.94	0.17
NP_001011901	Hsph1	heat shock protein 105 kDa	-0.91	0.14
NP_037036	Anxa1	annexin A1	-0.91	0.20
NP_001004223	Eef1g	elongation factor 1-gamma	-0.91	0.00
NP_001120766	Ndufb9	NADH dehydrogenase [ubiquinone] 1 beta subcomplex subunit 9	-0.90	0.32
NP_001014102	Uba1	ubiquitin-like modifier-activating enzyme 1	-0.90	0.08
NP_742005	Canx	calnexin precursor	-0.89	0.21
NP_112359	Rnpep	aminopeptidase B	-0.89	0.12
NP_072114	Tkt	transketolase	-0.89	0.19
NP_001103355	Fam129b	niban-like protein 1	-0.87	0.22
NP_058945	Esyt1	extended synaptotagmin-1	-0.86	0.02
NP_037184	Ywhah	14-3-3 protein eta	-0.86	0.15
NP_058710	Gstm1	glutathione S-transferase Mu 1	-0.86	0.30

Supplemental Table 1.
continued

RefSeq	Gene Symbol	Protein Name	Log2 (NK/Control) Mean	SE
NP_113698	Idh1	isocitrate dehydrogenase [NADP] cytoplasmic	-0.85	0.13
NP_596909	Slc25a10	mitochondrial dicarboxylate carrier	-0.85	0.28
NP_001013902	Isyna1	inositol-3-phosphate synthase 1	-0.84	0.20
NP_001098083	Hnrnpa2b1	heterogeneous nuclear ribonucleoproteins A2/B1	-0.84	0.26
NP_942040	Atp6v1e1	V-type proton ATPase subunit E 1	-0.83	0.19
NP_001099367	Cct8	T-complex protein 1 subunit theta	-0.82	0.24
NP_598302	Hadhb	trifunctional enzyme subunit beta, mitochondrial precursor	-0.82	0.27
NP_570090	Gnb2l1	guanine nucleotide-binding protein subunit beta-2-like 1	-0.82	0.02
NP_059002	Eci1	enoyl-CoA delta isomerase 1, mitochondrial precursor	-0.81	0.20
NP_001101978	Eps8l2	epidermal growth factor receptor kinase substrate 8-like protein 2	-0.81	0.19
NP_112297	Gnai2	guanine nucleotide-binding protein G(i) subunit alpha-2	-0.80	0.13
NP_036702	Glud1	glutamate dehydrogenase 1, mitochondrial precursor	-0.79	0.07
NP_001131115	Gstt3	glutathione S-transferase, theta 3	-0.79	0.24
NP_113791	Ywhae	14-3-3 protein epsilon	-0.77	0.26
NP_001101210	Ctnnd1	catenin delta-1	-0.77	0.27
NP_001014183	Idh2	isocitrate dehydrogenase [NADP], mitochondrial precursor	-0.77	0.18
NP_114039	Phb	prohibitin	-0.76	0.23
NP_058784	Gdi1	rab GDP dissociation inhibitor alpha	-0.75	0.22
NP_113863	Actn4	alpha-actinin-4	-0.75	0.08
NP_446428	Krt18	keratin, type I cytoskeletal 18	-0.75	0.17
NP_001094220	Suclg2	succinyl-CoA ligase [GDP-forming] subunit beta, mitochondrial	-0.75	0.11
NP_110463	Erap1	endoplasmic reticulum aminopeptidase 1 precursor	-0.75	0.21
NP_037023	Acadvl	very long-chain specific acyl-CoA dehydrogenase, mitochondrial precursor	-0.74	0.13
NP_955792	Krt19	keratin, type I cytoskeletal 19	-0.74	0.19
NP_001011995	Tuba1c	tubulin alpha-1C chain	-0.74	0.27
NP_647540	Ehd4	EH domain-containing protein 4	-0.74	0.20
NP_997475	Gpi	glucose-6-phosphate isomerase	-0.73	0.12
NP_001020868	Hsdl2	hydroxysteroid dehydrogenase-like protein 2	-0.73	0.24
NP_112355	Rala	ras-related protein Ral-A precursor	-0.73	0.20
NP_071985	Prdx3	thioredoxin-dependent peroxide reductase, mitochondrial precursor	-0.72	0.22
NP_037320	Pygb	glycogen phosphorylase, brain form	-0.71	0.12
NP_062040	Sptbn2	spectrin beta chain, non-erythrocytic 2	-0.71	0.04
NP_446041	Rab14	ras-related protein Rab-14	-0.69	0.04
NP_075237	Sfxn3	sideroflexin-3	-0.69	0.08

Supplemental Table 1.
continued

RefSeq	Gene Symbol	Protein Name	Log_2 (NK/Control) Mean	SE
NP_001007621	Pdhb	pyruvate dehydrogenase E1 component subunit beta, mitochondrial precursor	-0.67	0.06
NP_059055	Rab10	ras-related protein Rab-10	-0.67	0.15
NP_113902	Hrsp12	ribonuclease UK114	-0.66	0.23
NP_001012055	Cdh16	cadherin-16 precursor	-0.66	0.17
NP_062259	Tgm2	protein-glutamine gamma-glutamyltransferase 2	-0.66	0.21
NP_445859	Asah1	acid ceramidase precursor	-0.66	0.22
NP_077374	Aco2	aconitate hydratase, mitochondrial precursor	-0.65	0.03
NP_071852	Aldh1a1	retinal dehydrogenase 1	-0.65	0.07
NP_071794	Calr	calreticulin precursor	-0.63	0.17
NP_001100718	Vcl	vinculin	-0.63	0.15
NP_001099891	Tpd52	tumor protein D52	-0.63	0.04
NP_001006982	Dlst	dihydrolipoyllysine-residue succinyltransferase component of 2-oxoglutarate dehydrogenase complex, mitochondrial	-0.63	0.05
NP_001099959	Ndufs3	NADH dehydrogenase [ubiquinone] iron-sulfur protein 3, mitochondrial	-0.62	0.22
NP_446450	Rab8a	ras-related protein Rab-8A	-0.62	0.20
NP_062041	Arg2	arginase-2, mitochondrial precursor	-0.62	0.16
NP_150238	Mdh1	malate dehydrogenase, cytoplasmic	-0.61	0.04
NP_037035	Anp32a	acidic leucine-rich nuclear phosphoprotein 32 family member A	-0.61	0.16
NP_112643	Vdac1	voltage-dependent anion-selective channel protein 1	-0.61	0.15
XP_001059344	LOC684270	PREDICTED: isochorismatase domain-containing protein 2A, mitochondrial-like isoform 2	-0.60	0.19
NP_476489	Sept2	septin-2	-0.60	0.17
NP_775125	Tubb5	tubulin beta-5 chain	-0.59	0.18
NP_071795	Bcat2	branched-chain-amino-acid aminotransferase, mitochondrial precursor	-0.59	0.14
NP_058718	Itgb1	integrin beta-1 precursor	-0.58	0.17
NP_071785	Qdpr	dihydropteridine reductase	-0.58	0.09
NP_113906	Rab2a	ras-related protein Rab-2A	-0.58	0.07

Supplemental Table 2. A list of the significantly increased proteins in response to potassium depletion (*P*<0.05 by two tailed t-test and average $\log_2$(NK/Control) ≥ 0.58).

RefSeq	Gene Symbol	Protein Name	Log_2 (NK/Control)	
			Mean	SE
NP_058843	Cfl1	cofilin-1	1.43	0.43
NP_446083	Banf1	barrier-to-autointegration factor	1.30	0.42
XP_002727937	LOC100362689	PREDICTED: keratin 8-like	0.99	0.23

Supplemental Table 3. The over-represented GO clusters analyzed by the DAVID bioinformatics resources (using a list of all IMCD-expressed transcripts as a background).

Category	Term	No.	PValue	Genes	Fold Enrichment	Benjamini	FDR
Annotation Cluster 1	Enrichment Score: 3.0784975303793543						
GOTERM_BP_FAT	GO:0006091~generation of precursor metabolites and energy	17	5.19E-07	Uqcrfs1, Pygb, Atp5b, Dlst, Pfkl, Gpi, Suclg2, Atp6v1e1, Tpi1, Pgam1, Aco2, Mdh1, Pdhb, Atp6v1a, Idh2, Idh1, Atp5i	4.5831	0.0007	0.0009
GOTERM_BP_FAT	GO:0006084~acetyl-CoA metabolic process	7	8.83E-05	Suclg2, Aco2, Mdh1, Pdhb, Dlst, Idh2, Idh1	9.0820	0.0196	0.1446
GOTERM_BP_FAT	GO:0006099~tricarboxylic acid cycle	6	1.06E-04	Suclg2, Aco2, Mdh1, Dlst, Idh2, Idh1	11.8622	0.0202	0.1740
GOTERM_BP_FAT	GO:0046356~acetyl-CoA catabolic process	6	1.35E-04	Suclg2, Aco2, Mdh1, Dlst, Idh2, Idh1	11.3231	0.0224	0.2209
GOTERM_BP_FAT	GO:0009109~coenzyme catabolic process	6	1.35E-04	Suclg2, Aco2, Mdh1, Dlst, Idh2, Idh1	11.3231	0.0224	0.2209
GOTERM_BP_FAT	GO:0006732~coenzyme metabolic process	11	2.02E-04	Suclg2, Tpi1, Aco2, Tkt, Gstm1, Mdh1, Pdhb, Gstt3, Dlst, Idh2, Idh1	4.2682	0.0224	0.3304
GOTERM_BP_FAT	GO:0009060~aerobic respiration	6	2.57E-04	Suclg2, Aco2, Mdh1, Dlst, Idh2, Idh1	9.9643	0.0244	0.4204
GOTERM_BP_FAT	GO:0051187~cofactor catabolic process	6	3.12E-04	Suclg2, Aco2, Mdh1, Dlst, Idh2, Idh1	9.5810	0.0276	0.5105
Annotation Cluster 2	Enrichment Score: 2.272496263371351						
GOTERM_MF_FAT	GO:0019001~guanyl nucleotide binding	17	1.08E-05	Sept2, Gnai2, Rhoa, Glud1, Rab14, Rab2a, Tuba1c, Suclg2, Rab10, Eef2, Rala, Ehd2, Tgm2, Tubb5, Ehd4, Tuba4a, Rab8a	3.6442	0.0037	0.0147
GOTERM_MF_FAT	GO:0032561~guanyl ribonucleotide binding	17	1.08E-05	Sept2, Gnai2, Rhoa, Glud1, Rab14, Rab2a, Tuba1c, Suclg2, Rab10, Eef2, Rala, Ehd2, Tgm2, Tubb5, Ehd4, Tuba4a, Rab8a	3.6442	0.0037	0.0147
GOTERM_MF_FAT	GO:0005525~GTP binding	16	3.10E-05	Sept2, Gnai2, Rhoa, Glud1, Rab14, Rab2a, Tuba1c, Rab10, Eef2, Ehd2, Rala, Tgm2, Tubb5, Ehd4, Tuba4a, Rab8a	3.5399	0.0053	0.0423
GOTERM_MF_FAT	GO:0003924~GTPase activity	9	1.36E-04	Sept2, Rhoa, Eef2, Ehd2, Rab14, Tubb5, Ehd4, Tuba1c, Tuba4a	5.7285	0.0153	0.1848
GOTERM_BP_FAT	GO:0007264~small GTPase mediated signal transduction	9	0.0243	Iqgap1, Rab10, Ywhab, Rhoa, Rala, Gdi1, Rab14, Rab2a, Rab8a	2.5247	0.5122	33.1411
Annotation Cluster 3	Enrichment Score: 1.0300588015817522						
GOTERM_CC_FAT	GO:0005916~fascia adherens	3	0.0063	Vcl, Sptan1, Ctnnb1	22.9568	0.0890	7.9027
GOTERM_CC_FAT	GO:0014704~intercalated disc	3	0.0168	Vcl, Sptan1, Ctnnb1	14.3480	0.1683	19.7667
GOTERM_CC_FAT	GO:0005913~cell-cell adherens junction	4	0.0379	Vcl, Dlst, Sptan1, Ctnnb1	5.2774	0.2606	39.4641
GOTERM_CC_FAT	GO:0005912~adherens junction	6	0.0417	Flnb, Itgb1, Vcl, Dlst, Sptan1, Ctnnb1	3.1023	0.2624	42.5027
GOTERM_CC_FAT	GO:0070161~anchoring junction	6	0.0459	Flnb, Itgb1, Vcl, Dlst, Sptan1, Ctnnb1	3.0206	0.2787	45.7232
GOTERM_CC_FAT	GO:0044449~contractile fiber part	4	0.0487	Vcl, Krt19, Sptan1, Ctnnb1	4.7827	0.2862	47.7020
Annotation Cluster 4	Enrichment Score: 1.01236038334107						
GOTERM_BP_FAT	GO:0030036~actin cytoskeleton organization	10	2.34E-04	Itgb1, Rhoa, Cfl1, Actn4, Rala, Ehd2, Calr, Krt19, Sptan1, Ezr	4.6649	0.0239	0.3832
GOTERM_BP_FAT	GO:0030029~actin filament-based process	10	3.55E-04	Itgb1, Rhoa, Cfl1, Actn4, Rala, Ehd2, Calr, Krt19, Sptan1, Ezr	4.4168	0.0294	0.5800
GOTERM_BP_FAT	GO:0008064~regulation of actin polymerization or depolymerization	6	0.0011	Itgb1, Rhoa, Cfl1, Actn4, Rala, Ehd2, Calr, Krt19, Sptan1, Ezr	7.3267	0.0813	1.8431
GOTERM_BP_FAT	GO:0030832~regulation of actin filament length	6	0.0011	Rhoa, Cfl1, Actr3, Capg, Sptbn2, Sptan1	7.3267	0.0813	1.8431
GOTERM_BP_FAT	GO:0007010~cytoskeleton organization	12	0.0014	Itgb1, Rhoa, Cfl1, Actn4, Rala, Ehd2, Calr, Krt19, Tubb5, Sptan1, Ctnnb1, Ezr	3.1138	0.0840	2.2210
GOTERM_BP_FAT	GO:0051017~actin filament bundle formation	3	0.0080	Rhoa, Actn4, Ezr	20.7589	0.2643	12.2756
GOTERM_BP_FAT	GO:0007015~actin filament organization	4	0.0231	Rhoa, Cfl1, Actn4, Ezr	6.3874	0.5025	31.8214

Supplemental Table 4. A list of the significantly changed proteins and their estimated half-lives based on $t_{1/2}$ values at basal conditions of mouse collecting duct proteins as reported by Sandoval et al. (http://helixweb.nih.gov/ESBL/Database/ProteinHalfLives/index.html).

Gene Symbol	Protein Name	Mean Log_2(NK/Control)	Estimated Half-Life (Hours)
Arg2	arginase-2, mitochondrial precursor	-0.62	0.94
Aqp2	aquaporin-2	-0.49	9.25
Cdh16	cadherin-16 precursor	-0.66	13.86
Picalm	phosphatidylinositol-binding clathrin assembly protein	-1.1	14.53
Fermt2	fermitin family homolog 2	-2.07	18.17
Ehd4	EH domain-containing protein 4	-0.74	19.71
Gnai2	guanine nucleotide-binding protein G(i) subunit alpha-2	-0.8	19.99
Itgb1	integrin beta-1 precursor	-0.58	23.03
Fam129b	niban-like protein 1	-0.87	23.96
Atp6v1a	V-type proton ATPase catalytic subunit A	-2.52	24.15
Atp6v1e1	V-type proton ATPase subunit E 1	-0.83	24.2
Ezr	ezrin	-1.46	25.54
Rab8a	ras-related protein Rab-8A	-0.62	27.17
Anxa11	annexin A11	-1.03	27.49
Vcp	transitional endoplasmic reticulum ATPase	-0.94	27.59
Eps8l2	epidermal growth factor receptor kinase substrate 8-like protein 2	-0.81	28.66
Hsph1	heat shock protein 105 kDa	-0.91	28.84
Actn4	alpha-actinin-4	-0.75	30.68
Eif3a	eukaryotic translation initiation factor 3 subunit A	-1.07	31.74
Hsdl2	hydroxysteroid dehydrogenase-like protein 2	-0.73	32.41
Tuba1c	tubulin alpha-1C chain	-0.74	33.58
Tgm2	protein-glutamine gamma-glutamyltransferase 2	-0.66	33.99
Rab10	ras-related protein Rab-10	-0.67	34.44
Tuba4a	tubulin alpha-4A chain	-1.41	35.35
Iqgap1	ras GTPase-activating-like protein IQGAP1	-1.37	35.4
Vcl	vinculin	-0.63	36.23
Vps35	maternal embryonic message 3	-0.99	37.06
Isyna1	inositol-3-phosphate synthase 1	-0.84	37.07
Rab14	ras-related protein Rab-14	-0.69	37.2
Ndufb9	NADH dehydrogenase [ubiquinone] 1 beta subcomplex subunit 9	-0.9	37.53
Flnb	filamin-B	-1.03	37.58
Bcat2	branched-chain-amino-acid aminotransferase, mitochondrial precursor	-0.59	37.67
Kpnb1	importin subunit beta-1	-1.39	38.08
Pepd	xaa-Pro dipeptidase	-1.06	38.63
Gstt3	glutathione S-transferase, theta 3	-0.79	38.95
Cfl1	cofilin-1	1.43	39.05
Kif5b	kinesin-1 heavy chain	-0.95	39.07
Eef2	elongation factor 2	-1.01	39.26
Anxa1	annexin A1	-0.91	39.81
Ywhab	14-3-3 protein beta/alpha	-1.1	39.98
Rala	ras-related protein Ral-A precursor	-0.73	40.22
Rab2a	ras-related protein Rab-2A	-0.58	40.72
Etf1	eukaryotic peptide chain release factor subunit 1	-0.97	41.09

Supplemental Table 4.
continued

Gene Symbol	Protein Name	Mean Log_2(NK/Control)	Estimated Half-Life (Hours)
Capns1	calpain small subunit 1	-1.26	41.36
Calr	calreticulin precursor	-0.63	42.23
Hspa4	heat shock 70 kDa protein 4	-0.96	42.4
Cct7	T-complex protein 1 subunit eta	-1.14	42.74
Hrsp12	ribonuclease UK114	-0.66	43.09
Cct8	T-complex protein 1 subunit theta	-0.82	43.52
Psmc5	26S protease regulatory subunit 8	-1.04	44.18
Suclg2	succinyl-CoA ligase [GDP-forming] subunit beta, mitochondrial	-0.75	44.62
Gstm1	glutathione S-transferase Mu 1	-0.86	44.69
Slc25a10	mitochondrial dicarboxylate carrier	-0.85	45.01
Ndufs3	NADH dehydrogenase [ubiquinone] iron-sulfur protein 3, mitochondrial	-0.62	45.81
Pygb	glycogen phosphorylase, brain form	-0.71	46.16
Ywhah	14-3-3 protein eta	-0.86	46.55
Gdi1	rab GDP dissociation inhibitor alpha	-0.75	46.72
Pccb	propionyl-CoA carboxylase beta chain, mitochondrial precursor	-1.07	46.92
Pdhb	pyruvate dehydrogenase E1 component subunit beta, mitochondrial precursor	-0.67	47.58
Ndufa8	NADH dehydrogenase [ubiquinone] 1 alpha subcomplex subunit 8	-0.94	48.24
Krt18	keratin, type I cytoskeletal 18	-0.75	48.28
Tubb5	tubulin beta-5 chain	-0.59	48.84
Pfkl	6-phosphofructokinase, liver type	-1.56	49.25
Manba	beta-mannosidase precursor	-0.94	50.58
Gnb2l1	guanine nucleotide-binding protein subunit beta-2-like 1	-0.82	50.84
Uqcrfs1	cytochrome b-c1 complex subunit Rieske, mitochondrial	-1.06	51.11
Mdh1	malate dehydrogenase, cytoplasmic	-0.61	51.67
Cct4	T-complex protein 1 subunit delta	-1.67	51.78
Hexa	beta-hexosaminidase subunit alpha precursor	-0.99	52.94
Ywhae	14-3-3 protein epsilon	-0.77	53.02
Krt19	keratin, type I cytoskeletal 19	-0.74	53.43
Esyt1	extended synaptotagmin-1	-0.86	53.44
Nit2	omega-amidase NIT2	-0.97	54.54
Psma4	proteasome subunit alpha type-4	-1.7	56.34
Eef1g	elongation factor 1-gamma	-0.91	57.16
Hadhb	trifunctional enzyme subunit beta, mitochondrial precursor	-0.82	58.08
Actr3	actin-related protein 3	-1.52	58.27
Psmd12	26S proteasome non-ATPase regulatory subunit 12	-2.25	59.65
Atp5b	ATP synthase subunit beta, mitochondrial precursor	-0.99	60.38
Glud1	glutamate dehydrogenase 1, mitochondrial precursor	-0.79	60.4
Anp32a	acidic leucine-rich nuclear phosphoprotein 32 family member A	-0.61	61.15
Aifm1	apoptosis-inducing factor 1, mitochondrial precursor	-1.18	61.72
Pgam1	phosphoglycerate mutase 1	-1.11	62.58
Aco2	aconitate hydratase, mitochondrial precursor	-0.65	62.74
Eci1	enoyl-CoA delta isomerase 1, mitochondrial precursor	-0.81	63.73

Supplemental Table 4.
continued

Gene Symbol	Protein Name	Mean Log_2(NK/Control)	Estimated Half-Life (Hours)
Dlst	dihydrolipoyllysine-residue succinyltransferase component of 2-oxoglutarate dehydrogenase complex, mitochondrial	-0.63	64.59
Prdx3	thioredoxin-dependent peroxide reductase, mitochondrial precursor	-0.72	68.84
Qdpr	dihydropteridine reductase	-0.58	69.98
Tpi1	triosephosphate isomerase	-0.95	70.78
Aldh4a1	delta-1-pyrroline-5-carboxylate dehydrogenase, mitochondrial	-1.18	75.14
Phb	prohibitin	-0.76	75.47
Vdac1	voltage-dependent anion-selective channel protein 1	-0.61	76.69
Slc25a12	LOW QUALITY PROTEIN: calcium-binding mitochondrial carrier protein Aralar1	-1.98	77.48
Acadvl	very long-chain specific acyl-CoA dehydrogenase, mitochondrial precursor	-0.74	77.51
Tkt	transketolase	-0.89	81.32
Rnpep	aminopeptidase B	-0.89	85.54
Asah1	acid ceramidase precursor	-0.66	88.6
Iah1	isoamyl acetate-hydrolyzing esterase 1 homolog	-2.55	94.11
Idh2	isocitrate dehydrogenase [NADP], mitochondrial precursor	-0.77	97.41
Lrba	lipopolysaccharide-responsive and beige-like anchor protein	-1.14	113.71
Erap1	endoplasmic reticulum aminopeptidase 1 precursor	-0.75	337.24
Aadat	kynurenine/alpha-aminoadipate aminotransferase, mitochondrial	-4.81	N/A
COX2	cytochrome c oxidase subunit II (mitochondrion)	-2.32	N/A
Rhoa	transforming protein RhoA precursor	-2.3	N/A
Me1	NADP-dependent malic enzyme	-1.91	N/A
Arrb1	beta-arrestin-1	-1.64	N/A
Lgals3	galectin-3	-1.54	N/A
Akr1a1	alcohol dehydrogenase [NADP(+)]	-1.37	N/A
Ctnnb1	catenin beta-1	-1.21	N/A
Ahcyl1	adenosylhomocysteinase-like 1	-1.15	N/A
Ssr4	translocon-associated protein subunit delta precursor	-1.14	N/A
Comt	catechol O-methyltransferase	-1.08	N/A
Chdh	choline dehydrogenase, mitochondrial	-1.02	N/A
Dnpep	aspartyl aminopeptidase	-1.02	N/A
Ehd2	EH domain-containing protein 2	-1.01	N/A
Capg	macrophage-capping protein	-1	N/A
Atp5i	ATP synthase subunit e, mitochondrial	-0.98	N/A
Sptan1	spectrin alpha chain, non-erythrocytic 1	-0.96	N/A
Uba1	ubiquitin-like modifier-activating enzyme 1	-0.9	N/A
Canx	calnexin precursor	-0.89	N/A
Idh1	isocitrate dehydrogenase [NADP] cytoplasmic	-0.85	N/A
Hnrnpa2b1	heterogeneous nuclear ribonucleoproteins A2/B1	-0.84	N/A
Ctnnd1	catenin delta-1	-0.77	N/A
Gpi	glucose-6-phosphate isomerase	-0.73	N/A
Sptbn2	spectrin beta chain, non-erythrocytic 2	-0.71	N/A

Supplemental Table 4.
continued

Gene Symbol	Protein Name	Mean Log_2(NK/Control)	Estimated Half-Life (Hours)
Sfxn3	sideroflexin-3	-0.69	N/A
Aldh1a1	retinal dehydrogenase 1	-0.65	N/A
Tpd52	tumor protein D52	-0.63	N/A
LOC684270	PREDICTED: isochorismatase domain-containing protein 2A, mitochondrial-like isoform 2	-0.6	N/A
Sept2	septin-2	-0.6	N/A
Banf1	barrier-to-autointegration factor	1.3	N/A
LOC100362689	PREDICTED: keratin 8-like	0.99	N/A

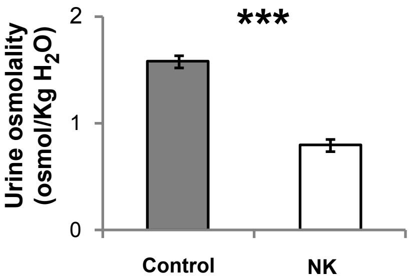

Supplementary Figure 1. Urine osmolality of urine recovered from bladders at 24 hours after potassium deprivation. Rats received a potassium-free diet (NK; □) or normal chow diet (control; ■) for 1 day. Upon sacrificing to harvest kidney tissues, urine was recovered from rats that had full bladders (n = 4 per each group). Bladder urine osmolality of potassium-deprived rats was significantly lower than that of control rats after 24 hours (0.8±0.1 vs. 1.6±0.06 osmol/kg H2O, respectively, P<0.01).

A

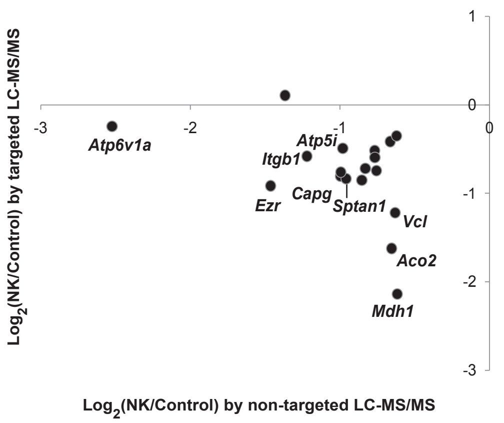

Protein Name	Gene Symbol	Log$_2$(NK/Control) targeted	Log$_2$(NK/Control) non-targeted
Integrin beta 1	*Itgb1*	-1.22	-0.58
Vinculin	*Vcl*	-1.22	-0.63
Ezrin	*Ezr*	-0.92	-1.46
Spectrin alpha chain, non-erythrocytic 1	*Sptan1*	-0.84	-0.96
Macrophage-capping protein	*Capg*	-0.81	-1
Alpha-actinin-4	*Actn4*	-0.74	-0.75
Catenin delta-1	*Ctnnd1*	-0.52	-0.77
Ras GTPase-activating-like protein IQGAP1	*Iqgap1*	0.11	-1.37
Malate dehydrogenase, cytoplasmic	*Mdh1*	-2.14	-0.61
Aconitate hydratase, mitochondrial precursor	*Aco2*	-1.62	-0.65
Isocitrate dehydrogenase [NADP] cytoplasmic	*Idh1*	-0.85	-0.85
Isocitrate dehydrogenase [NADP], mitochondrial precursor	*Idh2*	-0.59	-0.77
ATP synthase subunit beta, mitochondrial precursor	*Atp5b*	-0.76	-0.99
ATP synthase subunit e, mitochondrial	*Atp5i*	-0.49	-0.98
V-type proton ATPase subunit E 1	*Atp6v1e1*	-0.72	-0.83
V-type proton ATPase catalytic subunit A	*Atp6v1a*	-0.24	-2.52
NADH dehydrogenase [ubiquinone] iron-sulfur protein 7, mitochondrial	*Ndufs7*	-0.41	-0.66
NADH dehydrogenase [ubiquinone] iron-sulfur protein 3, mitochondrial	*Ndufs3*	-0.35	-0.62

B

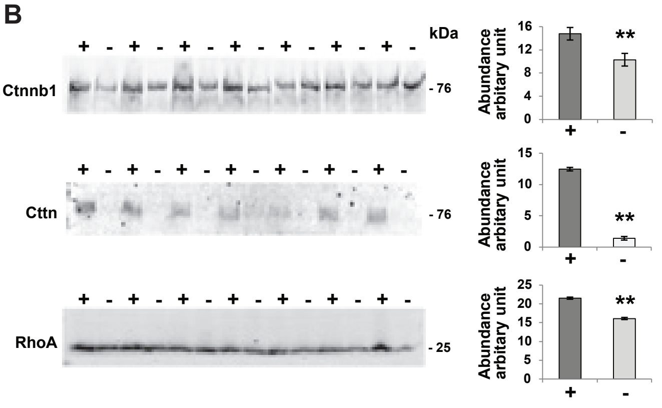

Supplementary Figure 2. Data verification by targeted LC-MS/MS and immunoblotting. (A) X and Y axes of the scatter plot represent protein abundance ratios [log2(NK/Control)] quantified by non-targeted and targeted LC-MS/MS techniques, respectively. (B) Immunoblotting of whole inner medullary proteins from rats fed with a potassium-free diet (-, n=7) and control rats (+, n=7) revealed significant down-regulation of Ctnnb1 (**, P<0.01), Cttn (***, P<0.005), and RhoA (*, P<0.05) in potassium-deprived (NK) rats (□) compared with control rats (■).

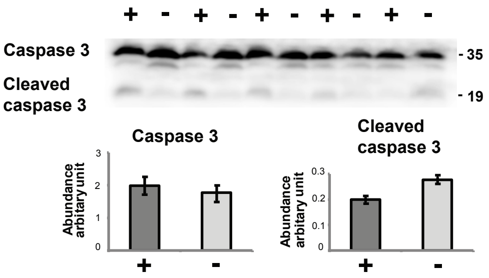

Supplementary Figure 3. Immunoblotting of whole inner medulla for caspase 3 and cleaved caspase 3. Rats received a potassium-free diet (-, n = 6) or normal chow diet (+, n = 6) for 1 day. The abundances of caspase 3 and cleaved caspase 3 in whole inner medulla were not significantly different between the 2 groups.

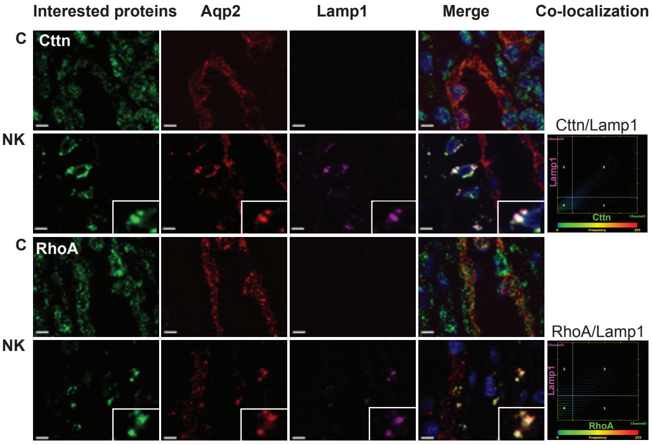

Supplementary Figure 4. Co-localization of other down-regulated proteins with autophagy markers. The inner medulla sections of control rats (C) and rats fed with a potassium-free diet for 1 day (NK) were triple-labeled against AQP2 (red), Lamp1 (pink), and down-regulated proteins including Cttn and RhoA (green). Insets demonstrate a magnified view of the areas where significant co-localization was observed as a group of puncta. These co-localized puncta were not observed in control sections. Scale bar = 4 µm. Scatter plots demonstrate the degree of colocalization between Cttn and Lamp1 (Pearson correlation coefficient, R = 0.80) as well as RhoA and Lamp1 (R = 0.74) in potassium-free condition.

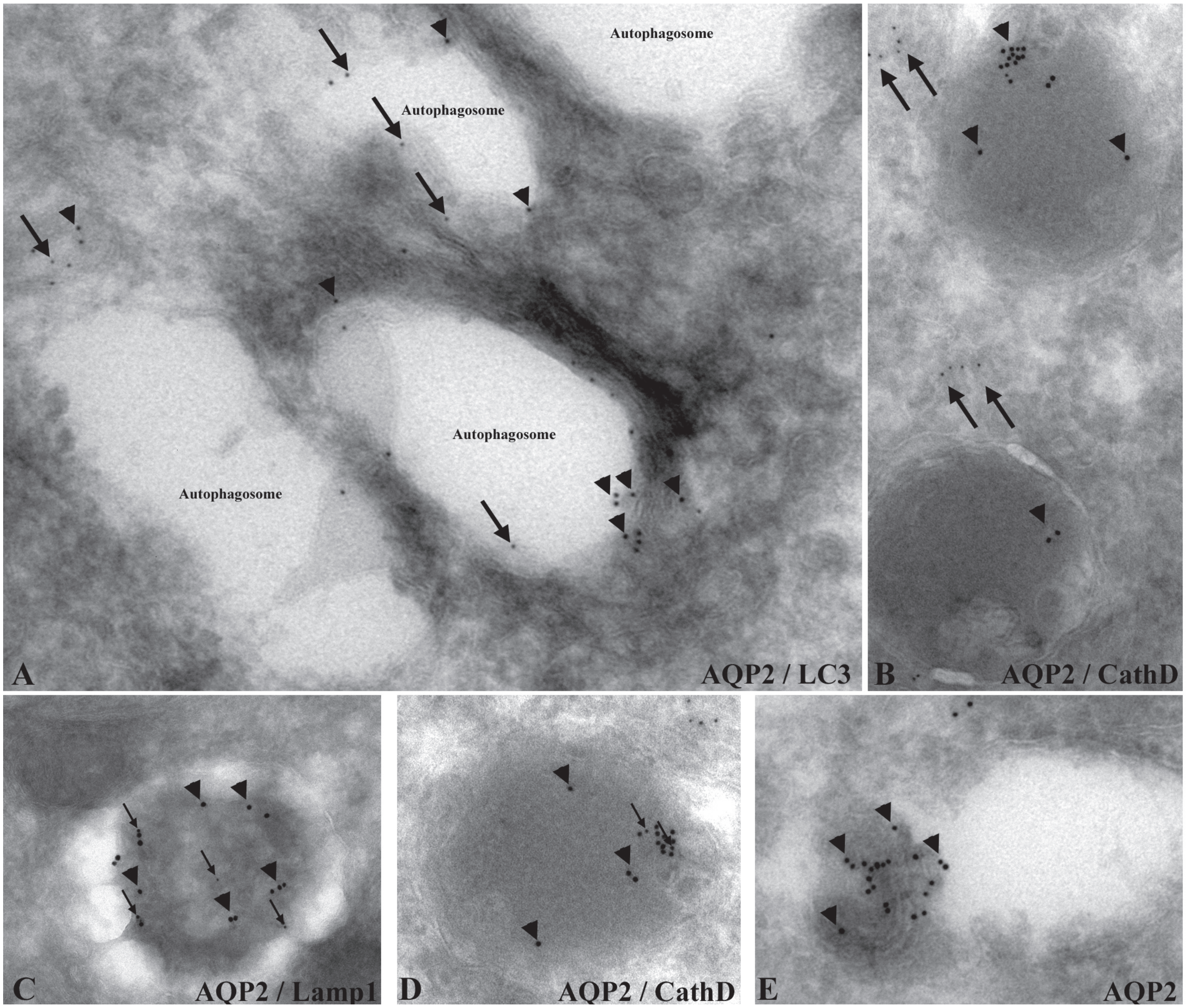

Supplementary Figure 5. Immunogold electron microscopy of AQP2 in autophagosomes. (A) In kidney inner medulla collecting ducts cells of potassium-deprived rats, multiple membrane-bound structures morphologically resembling autophagosomes were observed that co-labeled for AQP2 (small gold particles, arrows) and LC3β (large gold particles, arrow heads). B-E) Additional examples of AQP2 (small gold particles, arrows) in autophagosomes, where in the majority of instances it co-labeled with cathepsin-D or Lamp1 (large gold particles, arrow heads).

Supplementary Data 3. A list of protein names, peptide sequences, precursor m/z ratios, retention times, and integrated peak areas of the regulated proteins analyzed by the targeted LC-MS/MS.

Protein Name	Peptide Sequence	Sample	Charge	Precursor Mz	Retention Time	Area
sp|O88989|MDHC_RAT	EVGVYEALKDDSWLK	Control	2	876.45	37.19	27629933
sp|O88989|MDHC_RAT	EVGVYEALKDDSWLK	NK	2	876.45	37.12	8174179
sp|O88989|MDHC_RAT	GEFITTVQQR	Control	2	589.81	20.66	1.86E+09
sp|O88989|MDHC_RAT	GEFITTVQQR	NK	2	589.81	20.17	1.18E+09
sp|O88989|MDHC_RAT	NVIIWGNHSSTQYPDVNHAK	Control	3	760.71	23.25	2.68E+09
sp|O88989|MDHC_RAT	NVIIWGNHSSTQYPDVNHAK	NK	3	760.71	22.7	4.37E+08
sp|O88989|MDHC_RAT	VIVVGNPANTNCLTASK	Control	2	879.46	23.87	2.51E+09
sp|O88989|MDHC_RAT	VIVVGNPANTNCLTASK	NK	2	879.46	23.5	4.39E+08
sp|P10719|ATPB_RAT	AIAELGIYPAVDPLDSTSR	Control	2	994.52	40.91	53664217
sp|P10719|ATPB_RAT	AIAELGIYPAVDPLDSTSR	NK	2	994.52	40.87	2737559
sp|P10719|ATPB_RAT	DQEGQDVLLFIDNIFR	Control	2	961.49	73.66	80938848
sp|P10719|ATPB_RAT	DQEGQDVLLFIDNIFR	NK	2	961.49	73.79	85205822
sp|P10719|ATPB_RAT	FTQAGSEVSALLGR	Control	2	718.38	32.24	1.09E+08
sp|P10719|ATPB_RAT	FTQAGSEVSALLGR	NK	2	718.38	31.88	2.31E+08
sp|P10719|ATPB_RAT	VALTGLTVAEYFR	Control	2	720.40	41.92	66015491
sp|P10719|ATPB_RAT	VALTGLTVAEYFR	NK	2	720.40	42.1	34701128
sp|P10719|ATPB_RAT	VLDSGAPIKIPVGPETLGR	Control	3	640.37	32.59	3.67E+08
sp|P10719|ATPB_RAT	VLDSGAPIKIPVGPETLGR	NK	3	640.37	32.31	2.43E+08
sp|P10719|ATPB_RAT	VVDLLAPYAK	Control	2	544.82	29.96	2.44E+08
sp|P10719|ATPB_RAT	VVDLLAPYAK	NK	2	544.82	29.63	1.16E+08
sp|P15800|LAMB2_RAT	ALVEGGGILSR	Control	2	536.31	23.84	1.09E+09
sp|P15800|LAMB2_RAT	ALVEGGGILSR	NK	2	536.31	23.4	50355880
sp|P15800|LAMB2_RAT	DGFFGLSASNPR	Control	2	634.31	32	1.03E+08
sp|P15800|LAMB2_RAT	DGFFGLSASNPR	NK	2	634.31	31.67	67645938
sp|P15800|LAMB2_RAT	ETPSWTGVGFVR	Control	2	668.34	31.17	71017264
sp|P15800|LAMB2_RAT	ETPSWTGVGFVR	NK	2	668.34	30.94	39066516
sp|P15800|LAMB2_RAT	GFSGVFPACHPCHACFGDWDR	Control	3	827.34	30.77	26946637
sp|P15800|LAMB2_RAT	GFSGVFPACHPCHACFGDWDR	NK	3	827.34	30.46	13605183
sp|P15800|LAMB2_RAT	GSCYPATGDLLVGR	Control	2	733.36	26.7	2.01E+08
sp|P15800|LAMB2_RAT	GSCYPATGDLLVGR	NK	2	733.36	26.35	80190312
sp|P15800|LAMB2_RAT	KAEGVLAAQAR	Control	3	371.88	13.3	2.6E+08
sp|P15800|LAMB2_RAT	KAEGVLAAQAR	NK	3	371.88	12.85	67198128
sp|P15800|LAMB2_RAT	LGMVQAIVAAR	Control	2	564.83	29.19	1.2E+08
sp|P15800|LAMB2_RAT	LGMVQAIVAAR	NK	2	564.83	28.83	24492035
sp|P15800|LAMB2_RAT	LQELEGTYEENERELEVK	Control	3	736.69	25.94	1.48E+08
sp|P15800|LAMB2_RAT	LQELEGTYEENERELEVK	NK	3	736.69	25.39	42837940
sp|P15800|LAMB2_RAT	LREGQEVEFLVTSLPR	Control	3	625.01	37.43	47677949
sp|P15800|LAMB2_RAT	LREGQEVEFLVTSLPR	NK	3	625.01	37.41	12003665
sp|P15800|LAMB2_RAT	QKAETVQAALEEAQR	Control	3	557.96	22.04	1.43E+09
sp|P15800|LAMB2_RAT	QKAETVQAALEEAQR	NK	3	557.96	21.54	3.09E+08
sp|P15800|LAMB2_RAT	RLEQWAQELQQTGVLGAFESSFLNLQGK	Control	3	1,059.88	65.62	50843800
sp|P15800|LAMB2_RAT	RLEQWAQELQQTGVLGAFESSFLNLQGK	NK	3	1,059.88	65.93	45568353
sp|P15800|LAMB2_RAT	VLDISIPASPEQIQR	Control	2	833.46	33.73	1.13E+08
sp|P15800|LAMB2_RAT	VLDISIPASPEQIQR	NK	2	833.46	33.52	67180630
sp|P15800|LAMB2_RAT	YSEIEPSTEGEVIYR	Control	2	886.42	25.48	4.73E+08
sp|P15800|LAMB2_RAT	YSEIEPSTEGEVIYR	NK	2	886.42	25.07	75281209
sp|P15999|ATPA_RAT	AVDSLVPIGR	Control	2	513.80	25.05	2.06E+08
sp|P15999|ATPA_RAT	AVDSLVPIGR	NK	2	513.80	24.59	64808605
sp|P15999|ATPA_RAT	EVAAFAQFGSDLDAATQQLLSR	Control	3	780.06	62.07	2279068
sp|P15999|ATPA_RAT	EVAAFAQFGSDLDAATQQLLSR	NK	3	780.06	61.63	1634993
sp|P15999|ATPA_RAT	EVAAFAQFGSDLDAATQQLLSR	Control	2	1,169.59	62.06	2410507
sp|P15999|ATPA_RAT	EVAAFAQFGSDLDAATQQLLSR	NK	2	1,169.59	61.63	1502781
sp|P15999|ATPA_RAT	LKEIVTNFLAGFEP	Control	2	789.43	49.95	55017158
sp|P15999|ATPA_RAT	LKEIVTNFLAGFEP	NK	2	789.43	49.92	11788256
sp|P15999|ATPA_RAT	TGAIVDVPVGDELLGR	Control	2	805.94	37.62	4.02E+08
sp|P15999|ATPA_RAT	TGAIVDVPVGDELLGR	NK	2	805.94	37.63	3.58E+08
sp|P15999|ATPA_RAT	VVDALGNAIDGK	Control	2	586.32	22.9	2.72E+08
sp|P15999|ATPA_RAT	VVDALGNAIDGK	NK	2	586.32	22.38	1.21E+08

Protein Name	Peptide Sequence	Sample	Charge	Precursor Mz	Retention Time	Area
sp|P16086|SPTN1_RAT	ALINADELANDVAGAEALLDR	Control	2	1,077.56	59.87	11944585
sp|P16086|SPTN1_RAT	ALINADELANDVAGAEALLDR	NK	2	1,077.56	60.1	2228942
sp|P16086|SPTN1_RAT	DLASVQALLR	Control	2	543.32	36.68	82889968
sp|P16086|SPTN1_RAT	DLASVQALLR	NK	2	543.32	36.63	52267455
sp|P16086|SPTN1_RAT	DLSSVQTLLTK	Control	2	602.84	34.06	38216655
sp|P16086|SPTN1_RAT	DLSSVQTLLTK	NK	2	602.84	33.79	85398047
sp|P16086|SPTN1_RAT	KVEDLFLTFAK	Control	2	655.87	35.6	40655748
sp|P16086|SPTN1_RAT	KVEDLFLTFAK	NK	2	655.87	35.45	33799298
sp|P16086|SPTN1_RAT	LFGAAEVQR	Control	2	495.77	20.49	1.34E+09
sp|P16086|SPTN1_RAT	LFGAAEVQR	NK	2	495.77	19.98	2.18E+08
sp|P16086|SPTN1_RAT	LGESQTLQQFSR	Control	2	697.36	23	4.23E+08
sp|P16086|SPTN1_RAT	LGESQTLQQFSR	NK	2	697.36	22.47	79519088
sp|P16086|SPTN1_RAT	LQQSHPLSANQIQVK	Control	3	564.31	18.97	5.46E+08
sp|P16086|SPTN1_RAT	LQQSHPLSANQIQVK	NK	3	564.31	18.48	2.72E+08
sp|P16086|SPTN1_RAT	SSLSSAQADFNQLAELDR	Control	2	976.47	38.4	65671603
sp|P16086|SPTN1_RAT	SSLSSAQADFNQLAELDR	NK	2	976.47	38.32	53829417
sp|P25304|AGRIN_RAT	DQCPETCQFNSVCLSR	Control	2	1,000.92	25.45	2.96E+08
sp|P25304|AGRIN_RAT	DQCPETCQFNSVCLSR	NK	2	1,000.92	25.03	55283370
sp|P25304|AGRIN_RAT	SIESTLDDLFR	Control	2	648.33	49.28	8823450
sp|P25304|AGRIN_RAT	SIESTLDDLFR	NK	2	648.33	49.36	1085476
sp|P25304|AGRIN_RAT	SPVCGSDGVTYGTECDLKK	Control	3	691.65	19.22	5.93E+08
sp|P25304|AGRIN_RAT	SPVCGSDGVTYGTECDLKK	NK	3	691.65	18.8	1.4E+08
sp|P25304|AGRIN_RAT	VCTFGAVCSAGQCVCPR	Control	3	643.61	24.25	1.74E+08
sp|P25304|AGRIN_RAT	VCTFGAVCSAGQCVCPR	NK	3	643.61	23.92	22662303
sp|P25304|AGRIN_RAT	VCTFGAVCSAGQCVCPR	Control	2	964.92	24.25	5.65E+08
sp|P25304|AGRIN_RAT	VCTFGAVCSAGQCVCPR	NK	2	964.92	23.92	75280040
sp|P25304|AGRIN_RAT	VTCDGSYRPVCAQDGHTYNNDCWR	Control	3	977.74	20.58	1.82E+09
sp|P25304|AGRIN_RAT	VTCDGSYRPVCAQDGHTYNNDCWR	NK	3	977.74	20.04	7.93E+08
sp|P29419|ATP5I_RAT	ELAEAEDVSIFK	Control	2	675.84	30.58	34248784
sp|P29419|ATP5I_RAT	ELAEAEDVSIFK	NK	2	675.84	30.26	24347147
sp|P31977|EZRI_RAT	AKFYPEDVADELIQDITQK	Control	3	741.71	55.87	21598975
sp|P31977|EZRI_RAT	AKFYPEDVADELIQDITQK	NK	3	741.71	56.13	15579260
sp|P31977|EZRI_RAT	AQEEAERLEADR	Control	2	708.84	21.89	5.14E+08
sp|P31977|EZRI_RAT	AQEEAERLEADR	NK	2	708.84	21.35	2E+08
sp|P49134|ITB1_RAT	FCECDNFNCDR	Control	2	768.77	18.93	7.21E+08
sp|P49134|ITB1_RAT	FCECDNFNCDR	NK	2	768.77	18.49	3.44E+08
sp|P49134|ITB1_RAT	FQGPTCETCQTCLGVCAEHK	Control	3	795.00	21.18	1.36E+09
sp|P49134|ITB1_RAT	FQGPTCETCQTCLGVCAEHK	NK	3	795.00	20.74	5.24E+08
sp|P49134|ITB1_RAT	LRPEDITQIQPQQLLLK	Control	3	678.40	38.59	64676780
sp|P49134|ITB1_RAT	LRPEDITQIQPQQLLLK	NK	3	678.40	38.55	45004470
sp|P49134|ITB1_RAT	LRPEDITQIQPQQLLLK	Control	2	1,017.09	38.59	2143072
sp|P49134|ITB1_RAT	LRPEDITQIQPQQLLLK	NK	2	1,017.09	38.46	2955661
sp|P49134|ITB1_RAT	NVLSLTDRGEFFNELVGQQR	Control	3	774.73	41.69	29387689
sp|P49134|ITB1_RAT	NVLSLTDRGEFFNELVGQQR	NK	3	774.73	41.75	11240184
sp|P49134|ITB1_RAT	SAVTTVVNPK	Control	2	508.29	19.58	1.07E+09
sp|P49134|ITB1_RAT	SAVTTVVNPK	NK	2	508.29	22.9	1.79E+08
sp|P56574|IDHP_RAT	DLAGCIHGLSNVK	Control	2	692.36	26.47	1.5E+08
sp|P56574|IDHP_RAT	DLAGCIHGLSNVK	NK	2	692.36	26.02	16523431
sp|P56574|IDHP_RAT	GRPTSTNPIASIFAWTR	Control	3	625.67	20.87	1.98E+09
sp|P56574|IDHP_RAT	GRPTSTNPIASIFAWTR	NK	3	625.67	19.71	1.76E+09
sp|P56574|IDHP_RAT	LNEHFLNTTDFLDTIK	Control	3	640.99	39.82	1.12E+08
sp|P56574|IDHP_RAT	LNEHFLNTTDFLDTIK	NK	3	640.99	39.81	57352310
sp|P56574|IDHP_RAT	LNEHFLNTTDFLDTIK	Control	2	960.99	39.82	18298301
sp|P56574|IDHP_RAT	LNEHFLNTTDFLDTIK	NK	2	960.99	39.86	12129029
sp|P56574|IDHP_RAT	TIEAEAAHGTVTR	Control	2	678.35	15.17	5.53E+08
sp|P56574|IDHP_RAT	TIEAEAAHGTVTR	NK	2	678.35	14.64	4.85E+08
sp|P85972|VINC_RAT	AIPDLTAPVAAVQAAVSNLVR	Control	2	1,038.59	68.37	15602311
sp|P85972|VINC_RAT	AIPDLTAPVAAVQAAVSNLVR	NK	2	1,038.59	69	12632743
sp|P85972|VINC_RAT	AQQVSQGLDVLTAK	Control	2	729.40	25.3	58228975
sp|P85972|VINC_RAT	AQQVSQGLDVLTAK	NK	2	729.40	24.82	13247948
sp|Q6AYC4|CAPG_RAT	EGNPEEDITADQTNAQAAALYK	Control	3	783.70	27.26	59606240
sp|Q6AYC4|CAPG_RAT	EGNPEEDITADQTNAQAAALYK	NK	3	783.70	26.87	12313392
sp|Q6AYC4|CAPG_RAT	QAALQVADGFISR	Control	2	688.37	30.82	32302780
sp|Q6AYC4|CAPG_RAT	QAALQVADGFISR	NK	2	688.37	30.55	16019944

Protein Name	Peptide Sequence	Sample	Charge	Precursor Mz	Retention Time	Area
sp|Q6AYC4|CAPG_RAT	TTSGTTPAAIR	Control	2	538.29	17.7	1.31E+10
sp|Q6AYC4|CAPG_RAT	TTSGTTPAAIR	NK	2	538.29	17.29	9.43E+09
sp|Q6AYC4|CAPG_RAT	YREGGVESAFHK	Control	3	460.56	16.07	1.69E+08
sp|Q6AYC4|CAPG_RAT	YREGGVESAFHK	NK	3	460.56	15.49	1.11E+08
sp|Q6PCU2|VATE1_RAT	ARDDLITDLLNEAK	Control	3	529.62	42.43	24613327
sp|Q6PCU2|VATE1_RAT	ARDDLITDLLNEAK	NK	3	529.62	42.48	14961577
sp|Q99KI0|ACON_MOUSE	AKDINQEVYNFLATAGAK	Control	3	651.67	40.2	31959489
sp|Q99KI0|ACON_MOUSE	AKDINQEVYNFLATAGAK	NK	3	651.67	40.26	15263689
sp|Q99KI0|ACON_MOUSE	FKLEAPDADELPR	Control	3	500.93	27.79	23448224
sp|Q99KI0|ACON_MOUSE	FKLEAPDADELPR	NK	3	500.93	27.57	23901528
sp|Q99KI0|ACON_MOUSE	IVYGHLDDPANQEIER	Control	3	623.64	22.79	4.03E+09
sp|Q99KI0|ACON_MOUSE	IVYGHLDDPANQEIER	NK	3	623.64	22.22	8.89E+08
sp|Q99KI0|ACON_MOUSE	NAVTQEFGPVPDTAR	Control	2	801.40	25.13	7.64E+08
sp|Q99KI0|ACON_MOUSE	NAVTQEFGPVPDTAR	NK	2	801.40	24.66	1.62E+08
sp|Q9JKF1|IQGA1_MOUSE	EQLWLANEGLITK	Control	2	757.91	35.59	22087791
sp|Q9JKF1|IQGA1_MOUSE	EQLWLANEGLITK	NK	2	757.91	35.46	20840005
sp|Q9JKF1|IQGA1_MOUSE	ILAIGLINEALDEGDAQK	Control	2	942.01	48.9	5836516
sp|Q9JKF1|IQGA1_MOUSE	ILAIGLINEALDEGDAQK	NK	2	942.01	48.99	7172014
sp|Q9QXQ0|ACTN4_RAT	KAGTQIENIDEDFRDGLK	Control	3	683.68	27.13	12965927
sp|Q9QXQ0|ACTN4_RAT	KAGTQIENIDEDFRDGLK	NK	3	683.68	26.74	14027889
sp|Q9QXQ0|ACTN4_RAT	KDDPVTNLNNAFEVAEK	Control	3	635.32	30.06	22813940
sp|Q9QXQ0|ACTN4_RAT	KDDPVTNLNNAFEVAEK	NK	3	635.32	29.79	13642678
sp|Q9QXQ0|ACTN4_RAT	TIPWLEDRVPQK	Control	2	741.41	22.55	1.32E+09
sp|Q9QXQ0|ACTN4_RAT	TIPWLEDRVPQK	NK	2	741.41	22.06	3.61E+08
tr|D3ZG43|D3ZG43_RAT	FDLNSPWEAFPAYR	Control	2	856.91	45.78	8293906
tr|D3ZG43|D3ZG43_RAT	FDLNSPWEAFPAYR	NK	2	856.91	45.91	7244166
tr|D3ZG43|D3ZG43_RAT	SLADLTAVDVPTR	Control	2	679.37	30.64	73297380
tr|D3ZG43|D3ZG43_RAT	SLADLTAVDVPTR	NK	2	679.37	30.26	20075558
tr|D3ZG43|D3ZG43_RAT	VVAEPVELAQEFR	Control	2	743.90	34.06	45662024
tr|D3ZG43|D3ZG43_RAT	VVAEPVELAQEFR	NK	2	743.90	33.79	35823927
tr|D3ZZZ9|D3ZZZ9_RAT	ALSAIADLLTSEHER	Control	3	542.62	34.89	43370824
tr|D3ZZZ9|D3ZZZ9_RAT	ALSAIADLLTSEHER	NK	3	542.62	34.73	30304991
tr|D3ZZZ9|D3ZZZ9_RAT	ALSAIADLLTSEHER	Control	2	813.43	34.89	13136138
tr|D3ZZZ9|D3ZZZ9_RAT	ALSAIADLLTSEHER	NK	2	813.43	34.68	11156517
tr|D3ZZZ9|D3ZZZ9_RAT	GYELLFQPEVVR	Control	2	725.39	37.62	33706956
tr|D3ZZZ9|D3ZZZ9_RAT	GYELLFQPEVVR	NK	2	725.39	37.5	7987965
tr|D4A133|D4A133_RAT	AKEILQEEEDLAEIVQLVGK	Control	3	752.08	52.34	13329593
tr|D4A133|D4A133_RAT	AKEILQEEEDLAEIVQLVGK	NK	3	752.08	52.85	11274484
tr|F1LRD5|F1LRD5_RAT	FKDIFQEIYDK	Control	2	723.37	34.08	23763188
tr|F1LRD5|F1LRD5_RAT	FKDIFQEIYDK	NK	2	723.37	33.89	12406194
tr|F1LRD5|F1LRD5_RAT	LVTGWVKPIIIGR	Control	3	484.64	32.29	1.43E+08
tr|F1LRD5|F1LRD5_RAT	LVTGWVKPIIIGR	NK	3	484.64	32.02	75259744
tr|F1LRD5|F1LRD5_RAT	LVTGWVKPIIIGR	Control	2	726.46	32.29	54160062
tr|F1LRD5|F1LRD5_RAT	LVTGWVKPIIIGR	NK	2	726.46	32.02	31654584
tr|F1LRD5|F1LRD5_RAT	TVEAEAAHGTVTR	Control	2	671.34	12.74	3.82E+08
tr|F1LRD5|F1LRD5_RAT	TVEAEAAHGTVTR	NK	2	671.34	12.31	2.58E+08
tr|F1MAA7|F1MAA7_RAT	EAQLALGNAAADATEAK	Control	2	822.42	23.9	9.59E+08
tr|F1MAA7|F1MAA7_RAT	EAQLALGNAAADATEAK	NK	2	822.42	23.49	1.35E+08
tr|F1MAA7|F1MAA7_RAT	EGFFGNPLAPNPADK	Control	2	787.39	33.31	15994331
tr|F1MAA7|F1MAA7_RAT	EGFFGNPLAPNPADK	NK	2	787.39	33.1	15449792
tr|F1MAA7|F1MAA7_RAT	GKAEQQTADQLLAR	Control	3	510.27	19.12	8.23E+08
tr|F1MAA7|F1MAA7_RAT	GKAEQQTADQLLAR	NK	3	510.27	18.63	4.94E+08
tr|F1MAA7|F1MAA7_RAT	GKAEQQTADQLLAR	Control	2	764.91	19.08	6.06E+08
tr|F1MAA7|F1MAA7_RAT	GKAEQQTADQLLAR	NK	2	764.91	18.61	3.89E+08
tr|F1MAA7|F1MAA7_RAT	LCRPCQCNDNIDPNAVGNCNR	Control	3	849.69	18.47	8.05E+09
tr|F1MAA7|F1MAA7_RAT	LCRPCQCNDNIDPNAVGNCNR	NK	3	849.69	17.94	6.23E+09
tr|F1MAA7|F1MAA7_RAT	LGNTEACSPCHCSPVGSLSTQCDSYGR	Control	3	1,000.75	22.24	3.72E+09
tr|F1MAA7|F1MAA7_RAT	LGNTEACSPCHCSPVGSLSTQCDSYGR	NK	3	1,000.75	21.79	8.33E+08
tr|F1MAA7|F1MAA7_RAT	LNTFGDEVFNDPK	Control	2	748.36	28.36	1.17E+08
tr|F1MAA7|F1MAA7_RAT	LNTFGDEVFNDPK	NK	2	748.36	27.92	46524809
tr|F1MAA7|F1MAA7_RAT	LSAEDLVLEGAGLR	Control	2	721.90	41.37	2.54E+08
tr|F1MAA7|F1MAA7_RAT	LSAEDLVLEGAGLR	NK	2	721.90	41.37	94478398
tr|F1MAA7|F1MAA7_RAT	NTLQEANDILNNLKDFDR	Control	3	711.69	46.64	84195614
tr|F1MAA7|F1MAA7_RAT	NTLQEANDILNNLKDFDR	NK	3	711.69	46.67	50473072

Protein Name	Peptide Sequence	Sample	Charge	Precursor Mz	Retention Time	Area
tr|F1MAA7|F1MAA7_RAT	NTLQEANDILNNLKDFDR	Control	2	1,067.03	46.64	13721834
tr|F1MAA7|F1MAA7_RAT	NTLQEANDILNNLKDFDR	NK	2	1,067.03	46.67	9503503
tr|F1MAA7|F1MAA7_RAT	PSAYNFDNSPVLQEWVTATDIR	Control	3	841.74	51.44	27358551
tr|F1MAA7|F1MAA7_RAT	PSAYNFDNSPVLQEWVTATDIR	NK	3	841.74	51.66	27664903
tr|F1MAA7|F1MAA7_RAT	QDIAVISDTYFPR	Control	2	762.89	34.75	19701153
tr|F1MAA7|F1MAA7_RAT	QDIAVISDTYFPR	NK	2	762.89	34.54	18522501
tr|F1MAA7|F1MAA7_RAT	SAGYLDDVTLQSAR	Control	2	748.37	27.41	1.5E+08
tr|F1MAA7|F1MAA7_RAT	SAGYLDDVTLQSAR	NK	2	748.37	27.04	68896850
tr|F1MAA7|F1MAA7_RAT	SQECYFDPELYR	Control	2	803.85	30.93	76211584
tr|F1MAA7|F1MAA7_RAT	SQECYFDPELYR	NK	2	803.85	30.59	20130136
tr|F1MAA7|F1MAA7_RAT	SRVESTEQLIEIASR	Control	3	573.31	27.93	68400214
tr|F1MAA7|F1MAA7_RAT	SRVESTEQLIEIASR	NK	3	573.31	27.45	15200854
tr|F1MAA7|F1MAA7_RAT	SRVESTEQLIEIASR	Control	2	859.46	27.93	68054988
tr|F1MAA7|F1MAA7_RAT	SRVESTEQLIEIASR	NK	2	859.46	27.45	14960844
tr|F1MAA7|F1MAA7_RAT	TLPTGCFNTPSIEKP	Control	2	831.41	28.65	2.25E+08
tr|F1MAA7|F1MAA7_RAT	TLPTGCFNTPSIEKP	NK	2	831.41	28.4	1.09E+08
tr|Q5RJN0|Q5RJN0_RAT	AHQSVAATGSPSSTQSAVSK	Control	3	634.32	11.84	1.55E+08
tr|Q5RJN0|Q5RJN0_RAT	AHQSVAATGSPSSTQSAVSK	NK	3	634.32	11.38	1.13E+08
tr|Q5RJN0|Q5RJN0_RAT	LDDLINWAR	Control	2	558.30	33.36	12215269
tr|Q5RJN0|Q5RJN0_RAT	LDDLINWAR	NK	2	558.30	33.13	9494062
